# Chemistry and Pharmacology of Fluorinated Drugs Approved by the FDA (2016–2022)

**DOI:** 10.3390/ph16081162

**Published:** 2023-08-15

**Authors:** Ghulam Shabir, Aamer Saeed, Wajeeha Zahid, Fatima Naseer, Zainab Riaz, Nafeesa Khalil, Muneeba, Fernando Albericio

**Affiliations:** 1 Department of Chemistry, Quaid-I-Azam University, Islamabad 45320, Pakistan; shabirg@yahoo.com; 2 Department of Chemistry, Government Graduate College Toba Tek Singh, Punjab 36050, Pakistan; wajeehazahid86@gmail.com (W.Z.); fatimanaseer35@gmail.com (F.N.); zainabriaz49gb@gmail.com (Z.R.); nafeesakhalil20@gmail.com (N.K.); muneebahaleem515@gmail.com (M.); 3 School of Chemistry and Physics, University of KwaZulu-Natal, Durban 4001, South Africa; 4 CIBER-BBN, Networking Centre on Bioengineering, Biomaterials and Nanomedicine, Department of Organic Chemistry, University of Barcelona, 08028 Barcelona, Spain

**Keywords:** fluorinated drugs, radiolabeled fluoro-pharmaceuticals, fluorinated oligonucleotides, diverse biological activities

## Abstract

Fluorine is characterized by high electronegativity and small atomic size, which provide this molecule with the unique property of augmenting the potency, selectivity, metabolic stability, and pharmacokinetics of drugs. Fluorine (F) substitution has been extensively explored in drug research as a means of improving biological activity and enhancing chemical or metabolic stability. Selective F substitution onto a therapeutic or diagnostic drug candidate can enhance several pharmacokinetic and physicochemical properties such as metabolic stability and membrane permeation. The increased binding ability of fluorinated drug target proteins has also been reported in some cases. An emerging line of research on F substitution has been addressed by using ^18^F as a radiolabel tracer atom in the extremely sensitive methodology of positron emission tomography (PET) imaging. This review aims to report on the fluorinated drugs approved by the US Food and Drug Administration (FDA) from 2016 to 2022. It cites selected examples from a variety of therapeutic and diagnostic drugs. FDA-approved drugs in this period have a variety of heterocyclic cores, including pyrrole, pyrazole, imidazole, triazole, pyridine, pyridone, pyridazine, pyrazine, pyrimidine, triazine, purine, indole, benzimidazole, isoquinoline, and quinoline appended with either F-18 or F-19. Some fluorinated oligonucleotides were also authorized by the FDA between 2019 and 2022.

## 1. Introduction

**Figure 1:**
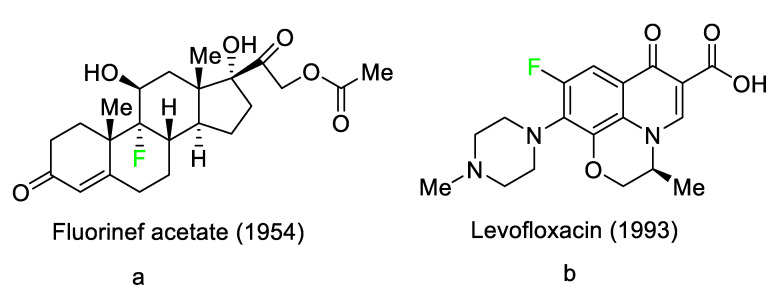
Selected fluoro-pharmaceuticals: (**a**) Fluorinef acetate, (**b**) Levofloxacin.

Organo-fluorinated drugs have been one of the most rapidly growing classes of organic compounds over the last 20 years [1,2,3,4,5,6,7,8,9,10,11]. The role of fluorine in the design of pharmaceutical drugs [12,13,14,15,16,17,18,19,20,21,22,23,24], agrochemicals, and specialty materials is widely recognized [25,26,27,28,29]. Fluoro-pharmaceuticals [3] are compounds of diverse nature containing at least one fluorine atom or a fluorinated functional group (e.g., trifluoromethyl, CF_3_). These pharmaceuticals have accounted for an estimated 20% of commercialized medications in recent years. Florinef acetate, the first fluoro-pharmaceutical, was introduced into the market in 1954 (Figure 1a). Florinef acetate is a corticosteroid synthesized with a fluorine atom in the stereogenic 9 position. It has strong mineralocorticoid properties as well as high glucocorticoid activity, and it is used to treat adrenogenital syndrome, adrenal insufficiency, and postural hypotension [30]. Fluoroquinolones (new quinolones) were released in the 1980s and they are another historically significant group of fluoro-pharmaceuticals (Figure 1b). Fluoroquinolones are effective antibacterial agents because they inhibit the activity of DNA gyrase and topoisomerase. This mechanism of action is fundamentally different from that of lactam antibiotics such as penicillin, cephalosporin, and antibacterial sulfa drugs. The number of fluoro-pharmaceuticals to receive FDA approval has continuously increased over the last two decades owing to the success of fluorinated corticosteroids and fluoroquinolones. Almost 300 fluoro-pharmaceuticals have been registered worldwide [30,31,32]. 

Several factors can explain the high occurrence of fluoro-organic molecules in medications. First and foremost, given that the fluorine (F, van der Waals radius of 1.47 Å) atom is slightly larger than the hydrogen (H, van der Waals radius of 1.20 Å) atom, it does not significantly alter the parent structure when H is replaced by F in a drug candidate [33]. Second, the C–F bond is the strongest link that carbon can make and it often boosts the metabolic stability of fluoro-pharmaceuticals. Third, as the most electronegative element (3.98), F causes bond polarization, which can affect the lipophilicity/hydrophilicity balance of a compound. Finally, F is a moderate hydrogen acceptor and the hydrogen bond-accepting analogy would imply a carbonyl moiety, which is especially pertinent given that the dipoles of C–F and C–O are sometimes comparable. Although the combination of these distinct features of F has a minor impact on the absorption, distribution, metabolism, and excretion of therapeutic candidates, these effects are likely to be complex and require further attention [34,35].

According to probability theory, the constant success of fluoro-pharmaceuticals implies that, for medicinal chemists, choosing fluoro-organic compounds is a viable method to considerably reduce the chance of unsuccessful trial-and-error attempts during drug discovery [36]. 

Drug discovery is a difficult, high-risk, expensive, and time-consuming process, with a success rate for small compounds estimated at 1/20,000 or 1/30,000 [36]. Although significant progress has been made in this field thanks to computer-aided approaches such as molecular modeling, these methods are still too immature for successful drug design, as evidenced by surprising failures in subsequent clinical phases [37].

In this review article, we profile the 60 fluorinated drugs approved by the FDA between 2016 and 2022 and discuss their structures, biological properties, distribution, and removal mechanisms for each one. 

## 2. FDA-Approved Fluorinated (F-18) Radiolabeled Drugs 

The development and progress of positron emission tomography (PET) imaging agents labeled with the radionuclide F-18 for uses spanning from fundamental research to clinical imaging have evolved rapidly. F-18 is the most appealing PET radionuclide for labeling small molecules because F can be easily incorporated without interfering with the biological activity of a compound, and the 110 min physical half-life of F-18 allows sufficient time for labeling, purification, quality control, and regional distribution to PET centers without on-site radiosynthetic capabilities. These ^18^F-labeled tracers have been utilized to image a variety of metabolic and biochemical processes related to cancer, Alzheimer’s disease, Parkinson’s disease, and cardiovascular illnesses. The most widely used radiopharmaceutical is ^18^F-fluoro deoxyglucose (FDG), which can indicate glucose absorption and energy use in diverse cells and has applications in oncology and neuroscience. FDG, like glucose, is taken up into cells by glucose transporters and is phosphorylated at the 1-position by hexokinase. However, after rapid phosphorylation, FDG-6-phosphate cannot be metabolized further because the F atom in position 2 prevents this, resulting in it being retained within the cell. Therefore, the intracellular FDG concentration is proportional to the glucose utilization of the tissue [38,39]. This is useful in oncology because tumor cells have a faster glycolytic rate and, hence, absorb FDG more actively than healthy cells.

FDG-PET can be used in clinical contexts such as initial diagnosis, assessment of response to therapy, and detection of recurrence. Lung nodules, non-small-cell lung carcinomas, lymphomas, melanomas, colorectal malignancies, and head and neck tumors are the tumor types most commonly studied with FDG-PET [40]. This technique has also been used in research and clinical practice to quantify cerebral glucose metabolism [41]. In clinical neurology, PET imaging can be used to perform differential diagnosis, define pathophysiological changes in the course of a disease, monitor illness, and also for follow-up purposes. FDG-PET can detect impaired glucose metabolism in a variety of disorders, including Alzheimer’s disease, Creutzfeldt–Jakob disease, and epilepsy [42]. Between 2016 and 2022, the FDA approved the following five fluorinated drugs containing F-18.

### 2.1. Flortaucipir F-18

Flortaucipir F-18 is a small lipophilic tracer capable of crossing the blood–brain barrier and binding to aggregated tau proteins. It is used in PET imaging for the diagnosis of Alzheimer’s disease (Table 1). Flortaucipir F-18 received medical approval for use in the US in May 2012. In the diagnosis of Alzheimer’s disease in adults, this radioactive tracer is used to image aggregated neurofibrillary tangles (NFTs) using PET. Patients who are receiving treatment for chronic traumatic encephalopathy should not use this drug. Flortaucipir F-18 rapidly penetrates the blood–brain barrier, circulates in the body, and binds to NFTs. After binding, the subsequent radioactive decay releases pairs of 511 keV gamma rays that are useful for imaging diagnostics. Alzheimer’s disease is diagnosed using the pattern and intensity of emission during imaging. The kidneys are the primary organs through which flortaucipir F-18 is removed. The original drug and four uncharacterized metabolites were first studied in mice. All metabolites were discovered in the kidneys and liver, except metabolite 2, which was detected only in the liver. Patients who overdose are more likely to experience severe side effects such as headaches and elevated blood pressure. It is advised to take symptomatic and supportive measures. Flortaucipir F-18 has an estimated half-life in plasma of 17.0 +/− 4.2 min [43]. 

### 2.2. Fluciclovine F-18

Fluciclovine F-18 is a synthetic amino acid that detects the upregulation of amino acid transport, especially in prostate cancer (Table 1). It is used for the identification of suspected sites of prostate cancer and it provides signals in 3 to 5 min.

Its miniature shape facilitates its uptake by tumor cells through its amino acid transporter without interacting with the body’s metabolic system. An individual suspected of having prostate cancer is expected to show an increased level of blood-specific prostate antigens. The overexpressed L-type amino acid transporters LAT1 and LAT3, which respond to binding with essential amino acids, play a significant role in the tumoral mechanisms of cell proliferation. Fluciclovine F-18 is transported into prostate cancer cells via ASCT1 (Alanine Serine Cysteine Transporter 1) and LAT1 (Large neutral amino acid transporter) injected intravenously. When an environment is acidic, the primary function of LAT1 is boosted. When injected into a human, fluciclovine F-18 takes roughly 2 to 10 min to reach the target cells. Additionally, within 90 min of the injection, a 63% reduction in mean tumor size is achieved. This drug is distributed throughout various organs such as the liver (14%), bone marrow (12%), lungs (7%), myocardium (4%), and pancreas (3%). The major characteristic of fluciclovine F-18 is that it is incapable of binding to plasma proteins and it does not participate in the synthesis of new proteins. Regarding its removal, 3% of the dose is eliminated through urine in the first four hours post-injection and 5% in the next twenty-four hours. One of the primary merits of fluciclovine F-18 is that it has the potential to be mutagenic but not carcinogenic [44]. 

### 2.3. Fluorodopa F-18

A fluorinated analogue of levodopa called fluorodopa F-18 is used in PET diagnostics to assess Parkinsonian disorders (Table 1). Used in conjunction with other diagnostic tests, fluorodopa F-18 PET is primarily utilized to visualize dopaminergic nerve terminals in the striatum. Aromatic amino acid decarboxylase (AADC) of the striatum converts fluorodopa F-18 to fluorodopamine F-18. Monoamine oxidase (MAO) continues to break down fluorodopa F-18 to produce ^18^F. Urine removes about 80% of the radiation that is injected. Following intravenous dosing, this drug has a plasma half-life of between 1 and 3 h [45]. 

### 2.4. Fluoroestradiol F-18

In May 2020, fluoroestradiol F-18 received FDA approval for use in patients with recurrent or metastatic breast cancer (Table 1). Fluoroestradiol F-18 is a radioactive diagnostic agent used in PET imaging for the detection of estrogen receptor-positive lesions as a supplement to biopsy. The drug can be absorbed by any tumor cell that expresses estrogen receptors, including those that originate in the uterus or the ovaries. In vivo ER expression can be measured using ^18^F-fluoroestradiol PET, and its effectiveness as a predictive assay and indicator of in vivo pharmacodynamic response to endocrine therapy has been established in clinical studies. Only 10% of the total activity of fluoroestradiol F-18 at 2 h after delivery is related to the parent drug, which is highly extracted and degraded by the liver. Urinary and biliary excretion are the main methods of elimination. Unbound metabolites, primarily glucuronide and sulphate conjugates, are released in the bile, reabsorbed by enterohepatic circulation, and subsequently eliminated through the kidneys [46].

### 2.5. Piflufolastat F-18

For the diagnosis of metastatic or recurring prostate cancer, piflufolastat F-18 is “a radiopharmaceutical diagnostic agent” used in PET to scan for PSMA-positive tumors (Table 1). Although the most frequent non-cutaneous tumor affecting men in North America is prostate cancer, visualizing the size and location of tumor metastases and recurrences remains an obstacle in the treatment of this disease. While MRI and CT scans provide more detail, PET images are more sensitive and can detect malignant tissue in any part of the body. Piflufolastat ^18^F, commonly known as ^18^F-DCFPyL, is a radiopharmaceutical made of urea that binds to PSMA and makes malignant prostate tissue visible. Under the trade name Pylarify, it received FDA approval for the first time in May 2021 to enable earlier and more precise diagnosis of probable prostate cancer metastases [47].

## 3. FDA-Approved Fluorinated Oligonucleotide Drugs

**Figure 2:**
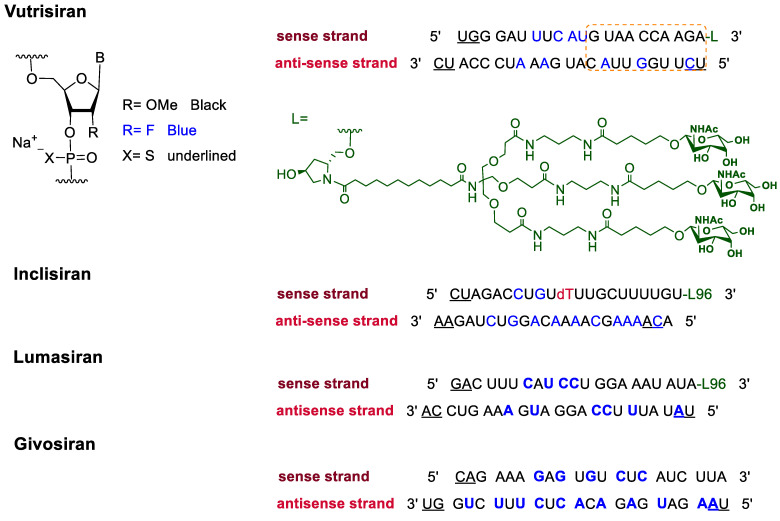
Structures of fluorinated oligonucleotides.

In addition to F-containing small molecules, in recent years, the FDA has approved (2019–2022) four oligonucleotides: vutrisiran (polyneuropathy of hereditary transthyretin-mediated amyloidosis in adults); inclisiran (atherosclerotic cardiovascular disease and familial hypercholesterolemia); lumasiran (hyperoxaluria type 1); and givosiran (acute hepatic porphyria). These four drugs share a similar structure (Figure 2): a double-stranded siRNA, with around 20 ribonucleosides for the sense and antisense strands (Table 2). They have a total of six thiophosphate linkages, as well as several 2′-F-ribonucleoside units to improve the stability of the double strand [48]. The remaining ribonucleosides are 2′-methoxy. With the same idea of increasing the stability, the 3′ end is linked to a short dendrimer of *N*-acetylgalactosamine (GalNAc) to mediate the binding and internalization of the drug by hepatocytes [49,50,51].

## 4. FDA-Approved Fluorinated Heterocyclic/Carbocyclic Drugs 

The presence of numerous heterocyclic rings in natural products such as alkaloids, vitamins, antibiotics, peptides, etc., prompted the introduction of these motifs in synthetic pharmaceuticals [52,53]. As a result, heterocycles are regarded important scaffolds for the synthesis of physiologically active molecules and potential drugs [54,55]. Pyrrole, pyrrolidine, pyrrolidine-2,5-dione, imidazole, pyrazole, triazole, pyridine, piperidine, pyrimidine, pyrazine, triazine, quinoline, indole, and associated analogues are the most frequent heterocyclic cores found in bioactive compounds. Indeed, heterocyclic moieties are found in around 85% of bioactive chemicals [56]. On the other hand, the inclusion of F atoms in pharmaceuticals introduced another essential tool for drug design in the second half of the twentieth century [57,58,59].

Since the introduction of the first fluorocorticosteroid, namely fludrocortisone, in 1954 [9], the fluorinated drug market has grown exponentially, with these medicines accounting for 20% of those on the market, and approximately 30% of them becoming blockbusters, such as Lipitor, Fluoxetine, Linezolid, and Fluticasone [59]. Over 300 fluorinated medicines have been approved for use as drugs to date [57]. The success of the introduction of F atoms is linked to the peculiar physicochemical properties of the C–F bond [60], namely its high bond strength, polarity, and minimal steric hindrance of F [61], combined with general metabolic stability, which is still being studied [62]. For example, the introduction of F allows researchers to readily modulate the pKa of neighboring functions, thereby enhancing bioavailability and affinity to certain receptors [63,64].

Furthermore, due to the high electronegativity of F, monofluorination or trifluoromethylation of alkyl groups reduces the lipophilicity of drugs. Fluoro-arenes, on the other hand, are more lipophilic due to the reduced polarizability of the C–F bond [65]. Moreover, the inclusion of an F atom improves membrane permeability [66]. Fluorinated drugs are especially important because they are used as diagnostic tools in imaging procedures such as ^19^F-MRI and ^18^F-PET [67,68,69], as mentioned in Section 2. Given the direct link between fluorinated moieties and heterocycles, a subclass of fluorinated heterocycles was established, which combines the strength of these two key scaffolds in modern medicinal chemistry. Fluorinated carbocyclic drugs are also available but these are in very small number as compared to fluorinated heterocyclic drugs.

The fluorinated heterocyclic/carbocyclic drugs approved by the FDA during 2016–2022 are reported in Table 3.

## 5. Therapeutic Indications

### 5.1. FDA-Approved Fluorinated Anticancer Drugs

F has perhaps never been more significant in medicinal chemistry than in the development of anticancer drugs. Isanbor and O’Hagan [122] reviewed fluorinated anticancer agents thoroughly, and Be’gue’ and Bonnet-Delpon [123] addressed fluorinated natural product anticancer agents. For cancer treatment, a growing number of fluorinated antimitotic/antitumor drugs are currently on the market [124,125,126,127]. 5-FU has been widely used to treat skin malignancies, as well as a wide range of solid tumors, including breast, colorectal, and stomach cancers. Fluorinated adenosine antimetabolites, such as fludarabine, clofarabine, and tezacitabine, are DNA polymerase inhibitors that can be used to treat a range of malignancies [128]. The following fluorinated drugs have recently been approved by the FDA.

#### 5.1.1. Abemaciclib

Abemaciclib was authorized by the FDA in September 2017 to treat metastatic breast cancer (Table 4). The cyclin-dependent kinases 4 and 6, which are involved in the cell cycle and promote the proliferation of cancer cells, are inhibited by abemaciclib. Endocrine therapy and chemotherapy were frequently used for treatment but they were ineffective for advanced cancer. Abemaciclib has been administered for the treatment of solid tumors, including glioblastoma, melanoma, and lymphoma, whether alone or in combination with fulvestrant. It continues to be beneficial for treatment of breast cancer in women who have HR-positive and HER2-negative hormone receptors. While immunotherapy using this drug takes 16 years to complete, the tumors of 19.7% of patients who take the medication totally or partially reduce in just 8 months. Following its elimination, 81% of the whole dose is found in feces and 3% in urine [70].

#### 5.1.2. Alpelisib 

The phosphatidylinositol 3-kinase (PI3K) inhibitor alpelisib shows strong anticancer efficacy (Table 4). It functions by inhibiting class I PI3K p110, the catalytic subunit of PI3K, a lipid kinase involved in several biological processes, such as cell proliferation, survival, differentiation, and metabolism. Alpelisib was developed to specifically target this enzyme, which appears to experience hyperactivation at a rate of approximately 30% in human malignancies. To treat postmenopausal women and men with advanced or metastatic breast cancer, alpelisib and fulvestrant are recommended. Additionally, adults and children over two years of age requiring systemic therapy and presenting severe PIK3CA-Related Overgrowth Spectrum (PROS) symptoms are treated with alpelisib. Based on reaction rate and duration, authorization of this indication was fast-tracked. A confirmation and description of clinical benefit in a confirmatory trial may be necessary for this indication to continue receiving approval. After two hours, the plasma concentration of alpelisib peaks at 1,320,912 ng/mL. The major metabolite of alpelisib is generated through hydrolysis processes. The metabolism of the drug is still unknown, but several processes have been postulated. The primary metabolic process results in the formation of the metabolite M4 or BZG791 by the replacement of an amine group on alpelisib with a hydroxyl group. Alpelisib can also be converted to the M1 and M12 metabolites via glucuronidation. A total amounting to 36% of an oral dose of this medication is excreted in an unmodified form in feces, and 32% is excreted in feces as the main metabolite BZG791. A dose taken orally is excreted in urine in two forms: 2.1% as the drug itself and 7.1% as the main metabolite BZG791; a total of 81% of an oral dose is ultimately excreted in feces, while 14% is excreted in urine. Alpelisib has an average half-life of 8–9 h. Patients exceeding recommended doses exhibit hyperglycemia, nausea, asthenia, and rashes [71].

#### 5.1.3. Apalutamide

Apalutamide, used to treat non-metastatic castration-resistant prostate cancer, contains trifluoromethyl pyridine and additional fluorophenyl functionalities (Table 4). An exposure–QT analysis of a 45-patient, open-label, uncontrolled, multi-center, single-arm dedicated QT study revealed the concentration-dependent lengthening of QTcF for apalutamide and its active metabolite. In mouse xenograft models of prostate cancer, apalutamide showed anticancer efficacy by reducing tumor volume and tumor cell growth. About 100% of the mean absolute oral bioavailability was recorded. Peak plasma concentration (T_max_) was reached, on average, in 2 h (range: 1 to 5 h). The C_max_ and AUC of apalutamide are anticipated to rise dose-proportionally. The average peak-to-trough ratio was 1.63, which indicates little daily variation in plasma concentrations of the drug. Following the suggested dosage, the main active metabolite *N*-desmethyl apalutamide had a steady-state C_max_ of 5.9 mcg/mL (1.0) and an AUC of 124 mcg/h/mL (23). Apalutamide had a mean apparent volume of distribution at steady-state of about 276 L. Protein binding, irrespective of concentration, for apalutamide was 96% and *N*-desmethyl apalutamide was 95% bound to plasma proteins. In patients with NM-CRPC, the mean effective half-life of apalutamide is approximately 3 days at steady state [129]. 

#### 5.1.4. Avapritinib

Avapritinib is a selective tyrosine kinase inhibitor that is being explored for the treatment of gastrointestinal cancers that are resistant to many drugs (Table 4). Platelet-derived growth factor receptor alpha (PDGFRA) exon 18 mutation is approved for the treatment of patients with metastatic or unresectable GIST. Ripretinib and avapritinib have a comparable mode of action. The transporters ABCB1 and ABCG2, which mediate the multidrug resistance phenotype of some malignancies, are negatively modulated by avapritinib. The interactions of this drug with the drug-binding pockets of these transporters may cause this modulation. Negative modulation of these transporters makes malignant cells more sensitive to paclitaxel and other chemotherapy drugs.

CYP3A4 and CYP2C9 are mainly responsible for avapritinib metabolism in vitro. Regarding elimination, 70% of avapritinib is eliminated in feces with 11% of the drug remaining unmodified, and 18% is eliminated in urine, with 0.23% of the drug remaining in its original form. Edema (swelling), nausea, asthenia (abnormal physical weakness or lack of energy), cognitive decline, vomiting, decreased appetite, diarrhea, hair color changes, increased lacrimation (tear secretion), abdominal pain, constipation, rash, and dizziness are common adverse effects of avapritinib. This drug has a half-life between 32 and 57 h [73].

#### 5.1.5. Belzutifan

The hypoxia-inducible factor 2 inhibitor belzutifan is used as an antineoplastic in the treatment of some tumors related to Von Hippel–Lindau (VHL) disease (Table 4). The HIF-2α protein was first identified in the 1990s by researchers at UT Southwestern Medical Center as a key player in the growth of certain cancers. Initially considered to be undruggable, a binding pocket was eventually discovered in the HIF-2α molecule, which allowed compounds to bind and inhibit these proteins. This discovery led to the initial development of belzutifan, which was further developed by a spin-off company named Peloton Pharmaceuticals, which itself was eventually acquired by Merck in 2019. In individuals with persistent systolic heart failure, vericiguat, a soluble guanylate cyclase activator, lowers the risk of cardiovascular death and heart failure-related hospitalization [76].

#### 5.1.6. Binimetinib

Binimetinib is a MEK inhibitor and a chemotherapy drug with anti-tumor properties (Table 4). At least 50% of the binimetinib dose was absorbed after oral administration in a pharmacokinetic trial, with a median time to maximum concentration (T_max_) of 1.6 h. In healthy subjects, binimetinib exposure was unaffected by the administration of a single dosage of MEKTOVI 45 mg (API binimetinib) with a high-fat, high-calorie meal (including roughly 150 calories from protein, 350 calories from carbohydrate, and 500 calories from fat) [78]. 

#### 5.1.7. Capmatinib 

Capmatinib obtained FDA approval in May 2020. Capmatinib is a kinase inhibitor used to treat non-small-cell lung cancer (NSCLC) with MET exon 14 skipping (Table 4). It targets the c-Met receptor tyrosine kinase. 

Capmatinib prevents c-Met-mediated phosphorylation of downstream signaling proteins as well as the proliferation and survival of c-Met-dependent tumor cells by inhibiting the phosphorylation of both wild-type and mutant variants of c-Met—a process triggered by the binding of its endogenous ligand, hepatocyte growth factor. The main metabolic pathways for capmatinib are CYP3A4 and aldehyde oxidase. Particular metabolic products and biotransformation processes remain to be clarified.

There is a lack of information about the toxicity and overdose of this drug. Both men and women using capmatinib should utilize effective contraception during treatment and for a week after stopping treatment due to evidence of embryo–fetal damage in animal studies. Capmatinib has an elimination half-life of 6.5 h [81].

#### 5.1.8. Dacomitinib

Dacomitinib was approved as a treatment for EGRF-mutated NSCLC (Table 4). Patients with NSCLC who have the EGFR exon 19 deletion or exon 21 L858R substitution mutations should receive dacomitinib as the first-line treatment according to an FDA-approved test volume of distribution. Dacomitinib has a 98% protein binding rate, and 79% of the dose provided is recovered in feces, of which 20% is an unmodified form, and 3% is excreted in urine. According to reports, dacomitinib has a relatively long half-life of 70 h [83]. 

#### 5.1.9. Encorafenib

Encorafenib is used to treat metastatic melanoma with certain mutations. Combined treatment with encorafenib and binimetinib is recommended for metastatic melanoma with a BRAF V600E or V600K mutation (Table 4). For the treatment of adult patients with metastatic colorectal cancer with a BRAF V600E mutation, encorafenib is also recommended in conjunction with cetuximab. The effectiveness of encorafenib in treating metastatic melanoma has been improved. The pharmacologic profile of a selective BRAF inhibitor (BRAFi) such as encorafenib differs from inhibitors of other clinically active BRAF genes. Encorafenib, a single drug, is administered once daily to patients with advanced or metastatic stages of melanoma. It has a distinct tolerability profile and varies in its anticancer efficacy. This drug has a median T_max_ of 2 h after oral treatment. At least 86% of the dosage is absorbed. The mean maximum concentration (C_max_) of encorafenib is reduced by 36% after a single dose of 100 mg BRAFTOVI (0.2 times the advised dose) was administered with a high-fat, high-calorie meal (roughly 150 calories from protein, 350 calories from carbohydrates, and 500 calories from fat). The apparent volume of distribution has a geometric mean (CV%) of 164 L. In vitro, 86% of encorafenib is bound to human plasma proteins. The drug has a median terminal half-life of 3.5 h [84].

#### 5.1.10. Ivosidenib

Ivosidenib is a first-generation inhibitor of isocitrate dehydrogenase-1 (IDH1) (Table 4). In some malignancies, IDH1 is frequently altered and overexpressed, which results in abnormal cell growth and proliferation. Ivosidenib blocks the activity of mutant IDH1, preventing cancer cells from differentiating further and from proliferating. Ivosidenib is an anticancer drug that works in malignancies with a sensitive IDH1 mutation, which shows elevated levels of the cancer cell oncometabolite D-2-hydroxyglutarate (D-2HG). By blocking IDH1, ivosidenib lowers D-2HG levels in a dose-dependent manner. This drug inhibits IDH1 in both its mutant and wild-type forms but not IDH2. Patients with recently diagnosed acute myeloid leukemia (AML) who were additionally treated with azacitidine had an apparent volume of distribution at a steady state of 504 L, while patients with relapsed or refractory AML had 403 L, and patients with cholangiocarcinoma had 706 L. Ivosidenib is 92–96% bound to plasma proteins in vitro. Patients with newly diagnosed AML who were additionally treated with azacitidine had a terminal half-life of 58 h at steady state, those with relapsed or refractory AML had a half-life of 98 h, and those with cholangiocarcinoma had a half-life of 129 h [91].

#### 5.1.11. Larotrectinib

Larotrectinib is used to treat solid tumors that have fusion genes for the neurotrophic receptor tyrosine kinase, are metastatic, high-risk for surgery, or have no other effective treatments (Table 4). Larotrectinib is a tropomyosin receptor kinase (Trk) inhibitor that can be taken orally, and it has anticancer properties. When larotrectinib is administered, it binds to Trk and prevents neurotrophin–Trk interaction and activation of Trk, which causes cellular apoptosis and inhibits cell proliferation in Trk-overexpressing tumors. Trk is a receptor tyrosine kinase that is triggered by neurotrophins. It is mutated in several types of cancer cells and it is crucial for the survival and proliferation of these cells. Larotrectinib does not cause any clinically significant QTc interval prolongation at doses nine times higher than the recommended adult dose. Following intravenous injection of healthy participants, larotrectinib has a recorded mean volume of distribution of 48 L. In vitro, larotrectinib binds to human plasma proteins with 70% affinity, independent of drug concentration. Its ratio of blood concentration to plasma concentration is 0.9. Larotrectinib has a half-life of 2.9 h [95]. 

#### 5.1.12. Lorlatinib

The FDA initially approved lorlatinib in November 2018 as a third-generation ALK tyrosine kinase inhibitor (TKI) for patients with metastatic NSCLC who have tested positive for ALK (Table 4). The *European Medicines Agency* (EMA) subsequently authorized it in 2019 for the treatment of a subset of patients with advanced NSCLC and this approval was later expanded in 2022 to include lorlatinib as a first-line treatment option. Following a single intravenous dose, the mean (CV%) steady-state volume of distribution (Vss) is 305 L (28%). At a concentration of 2.4 M in vitro, lorlatinib was 66% bound to plasma proteins with a blood-to-plasma ratio of 0.99 [96].

#### 5.1.13. Pexidartinib

Tenosynovial giant cell tumors (TGCT) are treated with pexidartinib which acts by blocking colony-stimulating factor 1 and its receptor (Table 4). The mean C_max_ and mean AUC following administration of single doses to healthy subjects and numerous doses to patients, respectively, are 8625 ng/mL and 77,465 ng.h/mL. The median T_max_ was 2.5 h, and it took about 7 days to reach a steady state. The C_max_ and AUC of pexidartinib were 100% higher when given with a high-fat meal, and its T_max_ was delayed by 2.5 h. Pexidartinib has an apparent volume of distribution of approximately 187 L. It enters the central nervous system in rats. It binds to serum proteins with 99% affinity, with 99.9% extensive binding to human serum albumin and 89.9% extensive binding to alpha-1-acid glycoprotein, according to the results of an in vitro plasma protein binding assay. Most of the oxidation and glucuronidation that pexidartinib undergoes is caused by hepatic CYP3A4 and UGT1A4. Pexidartinib is administered as a single dosage and after UGT1A4-mediated glucuronidation, a significant inactive *N*-glucuronide metabolite is generated with a 10% higher exposure than the parent drug. According to the results of in vitro research, CYP1A2 and CYP2C9 have a negligible impact on drug metabolism. Fecal excretion is the main route of pexidartinib elimination, accounting for 65% of its removal. Approximately 44% of the substance found in feces is recovered as the unmodified parent drug, and 27% of pexidartinib is eliminated through renal excretion, where more than 10% of the drug is detected as the *N*-glucuronide metabolite [130].

#### 5.1.14. Pralsetinib 

Pralsetinib is a kinase inhibitor with increased specificity for RET tyrosine kinase receptors (RTKs) over other RTK types (Table 4). NSCLC, among other tumors, is related to an increased expression of the RET oncogene. For the treatment of RET-driven malignancies, the first generation of targeted RET RTK inhibitors includes pralsetinib (BLU-667) and selpercatinib (LOXO-292).

In vitro, CYP3A4 is predominantly responsible for the metabolism of pralsetinib, with CYP2D6 and CYP1A2 having a minor role. Healthy volunteers who received a single oral dose of 310 mg of pralsetinib were found to have metabolites from oxidation (M453, M531, and M549b) and glucuronidation (M709) of the drug. With only a small amount of pralsetinib detected in urine (6%, 4.8% unmodified), this drug is predominantly removed through feces (73%, 66% unmodified). The risk of side effects such as musculoskeletal toxicity, exhaustion, constipation, hypertension, and pneumonia may rise with increased exposure [99].

#### 5.1.15. Relugolix 

Approved by the FDA in April 2022, relugolix is the first orally administered GnRH receptor antagonist for the treatment of prostate cancer. It has been demonstrated to outperform leuprolide, another androgen deprivation drug used to treat this condition (Table 4). 

Relugolix is recommended for the one-dose daily treatment of excessive menstrual bleeding associated with uterine fibroids in premenopausal women when taken in combination with estradiol and norethindrone. Luteinizing hormone (LH), which is produced in the pituitary gland after gonadotropin-releasing hormone (GnRH) binds to the appropriate GnRH receptors, stimulates the creation of testosterone in males in the Leydig cells of the testes. Relugolix inhibits the release of LH and, ultimately, testosterone by acting as a competitive antagonist of these GnRH receptors. The CYP3A subfamily of P450 enzymes metabolizes relugolix the most, with CYP2C8 contributing less. Approximately 81% of an oral dose is recovered in feces, of which 4.2% is the parent drug in its unmodified form, and 4.1% is excreted in urine, of which 2.2% is the parent drug in its unmodified form. Relugolix overdose information is not available, but it plays a significant impact in regulating reproductive processes by reducing the downstream generation of gonadal steroids. Relugolix has an average terminal elimination half-life of 60.8 h, while its average effective half-life is 25 h [103].

#### 5.1.16. Ripretinib 

Protein kinases are crucial for cellular activity, and their dysregulation can result in the development of cancer (Table 4). Most gastrointestinal stromal tumors are caused by protein kinases, particularly wild-type and mutant platelet-derived growth factor receptor A (PDGFRA) and KIT (GIST). Ripretinib has a distinctive dual mode of action that involves binding to both the activation loop and the kinase switch pocket, shutting off the kinase and its capacity to lead to dysregulated cell proliferation.

The active metabolite of ripretinib, DP-5439, is produced by the CYP3A subfamily of enzymes with assistance from CYP2D6 and CYP2E1. Like other kinase inhibitors, an overdose of ripretinib likely results in hematological toxicity, skin toxicity, as well as muscle, liver, and gastrointestinal toxicity. Ripretinib has an average half-life of 14.8 h [104].

#### 5.1.17. Rucaparib

Rucaparib is a poly (ADP-Ribose) polymerase (PARP) inhibitor used for high-grade cancers of the ovary, fallopian tubes, and peritoneum. It targets cancer cells with mutations in breast cancer (BRCA) genes (Table 4). An essential component of DNA repair is the enzyme PARP. Rucaparib specifically targets cancer cells with genetic mutations that lack a DNA repair mechanism and, thus, it kills cancer cells and slows tumor growth. It is administered orally twice a day with doses ranging between 240 and 840 mg. It reaches its target in 0 to 5.98 h [106].

#### 5.1.18. Selumetinib

Selumetinib received medical approval from the FDA in April 2020 and has been studied for the treatment of several types of cancer, including thyroid cancer and NSCLC (Table 4). Patients who take selumetinib undergo tumor shrinkage as a result of the suppression of drug ERK phosphorylation. Selumetinib is used to treat pediatric patients with neurofibromatosis type 1 who have symptomatic, inoperable plexiform neurofibromas. With an approximate population of 1/3000 people, NF-1 is regarded as rare. Mutations in the NF1 gene cause this genetic, autosomal dominant disorder, which may produce several difficulties, including the growth of many tumors in the neurological system. Selumetinib is excreted mainly in the feces (59%) and urine (33%). Selumetinib particularly inhibits subtypes 1 and 2 of the enzyme mitogen-activated protein kinase (MAPK kinase or MEK). These enzymes are a component of the MAPK/ERK pathway, which regulates cell division and growth and is overactive in many cancers. Selumetinib exerts its effects by specifically inhibiting MEK1 and MEK2. It decreases cell proliferation and stimulates pro-apoptotic signal transmission by blocking this oncogenic pathway. Selumetinib has a short half-life. In clinical practice, a dosage of 25 mg/m^2^ has an elimination half-life of 6.2 h.

The half-life varied from 9.2 to 10.6 h in studies exploring the pharmacokinetic consequences of several selumetinib regimens in selected Japanese patients. Selumetinib 75 mg was used twice daily in other studies, and the half-life was estimated to be around 13 h. Patients who receive an overdose are more likely to have side effects such as cardiomyopathy, eye toxicity, and diarrhea. Dialysis is generally believed to be ineffective in cases of overdose since selumetinib has protein binding ability [107].

#### 5.1.19. Sotorasib 

The KRAS inhibitor sotorasib, also referred to as AMG-510, is used to treat adult patients with NSCLC harboring the KRAS G12C mutation (Table 4). KRAS was first identified in 1982 but was not thought to be a druggable target until the mid-2010s. Sotorasib is the first KRAS inhibitor and is under investigation [108].

#### 5.1.20. Talazoparib

The FDA approved talazoparib in October 2018, under the brand name Talzenna 3, for the treatment of germline BRCA-mutant, HER2-negative, locally advanced, or metastatic breast cancer (Table 4). The authorization of Talzenna was based on the findings of the EMBRACA trial, which showed that talazoparib had a mean 8.6-month progression-free survival period compared to the physician’s choice of chemotherapy with a 5.6-month progression-free survival period. In cancer cells, talazoparib inhibits PARP from repairing DNA damage, thereby leading to accumulated damage and the build-up of PARP–DNA complexes. The cytotoxicity of talazoparib may also be influenced by repair-related errors made by secondary repair pathways that are prone to error. In 1–2 h, Talzenna pills attain their peak plasma concentration. When consumed with a high-fat meal, C_max_ drops by 43%, T_max_ increases by 1–4 h, but the AUC remains the same. The mean apparent volume of distribution for talazoparib is 420 L. The plasma protein binding of talazoparib, which is 74%, is unaffected by drug concentration [110].

### 5.2. FDA-Approved Fluorinated Anti-Viral Drugs 

Major developments in the treatment of various viral diseases have been made over the last two decades. However, in recent years, there has been an explosion of new viral diseases for which there are no effective treatments. Ebola, Zika, and novel strains of hepatitis and herpes viruses have spread, raising the possibility of a pandemic. There are three types of viral infections. The first class of viruses comprises life-threatening chronic viruses such as HIV, hepatitis B (HBV), and hepatitis C (HCV). Acute viral infections, such as influenza, fall into the second category since they are often nonlethal and self-resolving. The third type includes nonlethal viral illnesses (common cold caused by rhinoviruses) that have a considerable economic impact [97]. F-containing medicines, such as Paxlovid, the first oral tablet for the treatment of COVID-19, are a key subset of these antivirals. As of March 2022, over ten types of fluorinated anti-COVID-19 medicines had been documented. The addition of F is crucial since it increases the selectivity of drugs, allows them to dissolve in lipids, and slows the rate at which they metabolized, giving them more time to exert their effects. The following anti-viral F-containing drugs have been developed in response to the dramatic increase in novel viral diseases in recent years:

#### 5.2.1. Baloxavir Marboxil

Baloxavir marboxil is a first-in-class cap-dependent endonuclease inhibitor used to treat influenza (Table 5). It is a prodrug of baloxavir with a better absorption profile than its active metabolite as a result of the structural addition of a phenolic hydroxyl group. In a polymerase acidic (PA) endonuclease assay, it has an inhibitory concentration (IC_50_) of 1.4 to 3.1 nM for influenza A viruses and 4.5 to 8.9 nM for influenza B viruses. In influenza and avian influenza A murine models, baloxavir decreases lung viral loads and increases mouse survival rates. Within 24 h of dosing, a dose-dependent decrease in virus titer is observed. Human serum proteins are 92.9–93.9% bound to the active metabolite, baloxavir. The drug has an apparent terminal elimination half-life of 79.1 h [115].

#### 5.2.2. Fostamatinib

Fostamatinib is a spleen tyrosine kinase inhibitor used to treat chronic immune thrombocytopenia (Table 5). Due to its ability to affect the SYK inhibitor, fostamatinib has recently been recognized as a potential therapy for acute respiratory distress syndrome (ARDS) in patients with severe COVID-19. R406 has an apparent oral volume of distribution of 400 L. Plasma proteins are 98.3% bound to fostamatinib’s active metabolite R406. R406 has an elimination half-life of 15 h [131].

#### 5.2.3. Glecaprevir 

Glecaprevir is an HCV inhibitor and antiviral agent that prevents viral RNA replication and virion assembly (Table 5). It may be an effective treatment option for patients who are unable to benefit from treatment with other non-structural protein inhibitors. For the treatment of chronic hepatitis C virus of all genotypes without liver disease or mild liver inflammation, the FDA authorized the fixed-dose combination medication of pibrentasvir and glecaprevir in August 2017. A long-term hepatitis C virus infection may result in liver failure and impaired liver function, both of which have a detrimental effect on the quality of life of patients. The major objective of this pharmacological combination therapy is to cure patients while achieving an enhanced response in virion suppression. This medication does not participate in any other metabolic process. It interacts only with blood plasma proteins and it is eliminated through the renal tubular system (biliary–fecal). Studies conducted in vitro have found no evidence of genotoxic effects during mating, female or male fertility, or embryo development in rodents [132].

#### 5.2.4. Letermovir

Approved by the FDA in 2017, letermovir is used to treat cytomegalovirus (CMV) infection and also to prevent infection following bone marrow transplant (Table 5). A patient whose stem cells have been damaged by radiation and chemotherapy obtains healthy blood-forming stem cells from a donor. The new stem cells occasionally bond with proteins of new organisms after the transplant. In some instances, mutations occur, disrupting the ultrastructure of the genome and as a result, new, live viral particles are generated [77]. 

#### 5.2.5. Osilodrostat

Osilodrostat is recommended for the treatment of adult patients with Cushing’s disease for whom surgery to remove the pituitary is not an option or has not been effective (Table 5). Cushing’s disease is an endocrine illness brought on by prolonged and excessive exposure to glucocorticoids. A less frequent cause of Cushing’s disease is typically brought on by an excess release of the hormone adrenocorticotropic hormone (ACTH) from a pituitary adenoma.

The symptoms of this disease include muscle weakness, osteoporosis, thinning hair and skin, weight gain, and a variety of mental, cardiovascular, and immune abnormalities. Osilodrostat inhibits aldosterone synthase and 11-hydroxylase CYP11B1 to a lesser extent CYP11B2. Osilodrostat helps to restore endogenous cortisol levels and reduce the symptoms of Cushing’s disease by blocking CYP11B1, an enzyme that catalyzes the last step of cortisol production.

Osilodrostat metabolism is mediated by several CYP enzymes (CYP3A4, CYP2B6, and CYP2D6) and UDP glucuronosyl transferases, with no single enzyme accounting for more than 25% of the total clearance. It is not anticipated that the metabolites influence the pharmacological action of osilodrostat. After oral administration of radiolabeled osilodrostat, 90.6% of the radioactivity is excreted in urine and only 1.58% in feces. About 5.2% of the administered dose is excreted in the urine as the parent form, indicating that osilodrostat is cleared mainly by metabolism and urinary elimination.

Osilodrostat overdose can cause severe hypocortisolism since it inhibits cortisol production. Nausea, vomiting, exhaustion, hypotension, abdominal pain, loss of appetite, dizziness, and syncope are possible symptoms. Osilodrostat has a half-life of approximately 4 h [93].

#### 5.2.6. Pibrentasvir

Pibrentasvir is an HCV inhibitor and antiviral agent that prevents viral RNA replication and virion assembly (Table 5). It has proved to be an effective treatment option for patients for whom treatment with other non-structural protein inhibitors has been unsuccessful. In August 2017, the FDA authorized the fixed-dose combination medication of pibrentasvir and glecaprevir for the treatment of chronic HCV of all genotypes without liver disease or mild liver inflammation, including patients with severe renal disease who are regularly dialyzed. The major objective of this pharmacological combination therapy is to cure the patient while achieving an increased virion suppression response. Viral protease gene replication, assembly, and maturation are significantly influenced by non-structural proteins. Pibrentasvir prevents replication and disrupts assembly. This drug does not participate in any other metabolic process. It interacts only with blood plasma proteins and it is eliminated through the renal tubular system (biliary–fecal). Studies conducted in vitro have found no evidence of genotoxic effects during mating, female or male fertility, or embryo development in rodents [132].

#### 5.2.7. Siponimod

Siponimod is used to treat multiple sclerosis and it reduces the peripheral blood lymphocyte count in a dose-dependent manner (Table 5). When siponimod is used, it temporarily lowers heart rate and atrioventricular conduction and, in the first 6 h following intake, the greatest decrease in heart rate is observed. After oral administration of immediate-release oral dosages of siponimod, the time (T_max_) to reach maximum plasma concentrations (C_max_) is around 4 h (within a range of 3–8 h). Siponimod has a total oral bioavailability of roughly 84%. Steady-state concentrations of this drug are reached after approximately 6 days of daily treatment with a single dose. Food consumption delays the absorption of siponimod (the median T_max_ increases by approximately 2–3 h). Eating has no impact on siponimod exposure throughout the body. Siponimod has an average volume of distribution of 124 L. According to animal models, siponimod easily crosses the blood–brain barrier.

Large amounts of siponimod are metabolized, mostly via the enzymes CYP2C9 (79.3%) and CYP3A4 (18.5%). The clinical efficacy and safety of siponimod in humans are not expected to be caused by the pharmacological activity of the major metabolites M3 and M17. The main mechanisms by which siponimod is removed from systemic circulation include metabolism and subsequent biliary/fecal excretion [94].

#### 5.2.8. Sofosbuvir

Sofosbuvir is used to treat HCV in both adults and children older than 3 years (Table 5). It can be used in conjunction with other antiviral medications. To treat a particular HCV genotype, sofosbuvir is coupled with several additional antiviral medicines to create novel derivatives. Oral administration of this drug results in blood plasma concentrations of 567 ng/mL within 0.2 to 2 h. Nearly 61–65% of the dosage binds to human plasma proteins as soon as it enters the blood. It is eliminated via three routes: urine (80%), feces (14%), and respiration (2.5%). When used alone, it can have mild toxic effects like weariness and headaches [98].

#### 5.2.9. Tecovirimat

Tecovirimat is prescribed for the treatment of smallpox in adults and in children weighing at least 3 kg (Table 5). It inhibits the orthopoxvirus VP37 envelope-wrapping protein. Patients with immune deficiencies may experience a decrease in the effectiveness of this drug. In Europe, after receiving a smallpox vaccination, it is also recommended to treat issues brought on by the replication of the vaccinia virus. Tecovirimat is also used in Europe to treat adult and pediatric cases of cowpox and monkeypox. Tecovirimat is an antiviral medication that lowers viremia and helps to halt the transmission of viruses. All orthopoxviruses examined in vitro, including variola and the smallpox virus, are resistant to this drug. After oral administration of 600 mg of tecovirimat and intravenous administration of 200 mg of tecovirimat, this drug shows a volume of distribution of 383 L and 1030 L, respectively. Human plasma proteins are 77–82% bound to tecovirimat. Following intravenous injection of 200 mg of tecovirimat and oral administration of 600 mg of tecovirimat, the elimination half-life (CV%) is 21 h (45%) and 19 h (29%), respectively [102]. 

#### 5.2.10. Voxilaprevir

Voxilaprevir is a non-structural protein-containing drug used to treat adult liver disorders. It can be used in conjunction with sofosbuvir to treat chronic hepatitis C. The FDA authorized this medication in July 2017 (Table 5). Voxilaprevir can be used to treat infections with genotypes 1, 2, 3, 4, 5, and 6. HCV in the body begins to kill white blood cells (WBC) and, thus, causes the immune system to become inactive. HCV uses the human genome as a host to produce single-stranded RNA as a means of self-replication. Voxilaprevir directly destroys the targeted viral non-structural proteins, thus preventing HCV replications and reducing the amount of RNA in the replication complex [111].

#### 5.2.11. Umbralisib

Umbralisib is used to treat uncommon types of refractory lymphoma (Table 5). A rare, slowly spreading form of non-Hodgkin lymphoma, marginal zone lymphoma, is initially treated with rituximab (an anti-CD20 medication), either alone or in conjunction with chemotherapy. Unfortunately, many individuals relapse or become resistant to these medications. Rituximab and other chemotherapeutic drugs are also used to treat follicular lymphoma. Based on encouraging outcomes from clinical trials, the FDA approved the drug in February 2021. It has been authorized for the treatment of recurrent and refractory marginal cell lymphoma and follicular lymphoma in adults. In contrast to previous lymphoma therapies, umbralisib inhibits casein kinase, a key regulator of protein translation kinase-1 [112].

### 5.3. FDA-Approved Fluorinated Anti-HIV Drugs

Some novel F-containing compounds with considerable antiviral activity have been synthesized. Fluorinated purine nucleotides and nucleosides show strong antiviral (anti-human immunodeficiency virus (anti-HIV)) activity [116]. F has been added to the base as well as the sugar residue. F-containing antimetabolites, such as alovudine, the direct fluorine analogue of zidovudine (3-azidothymidine, AZT), are of interest in the development of anti-HIV medications. In cells, alovudine is phosphorylated to 5-triphosphate, an active inhibitor of HIV-associated reverse transcriptase [122]. The FDA recently approved emtricitabine ((-)-beta-L-2′,3′-dideoxy-5-fluoro-3′-thiacytidine) for the treatment of HIV. The following anti-HIV drugs have recently been approved by the FDA:

#### 5.3.1. Bictegravir

Bictegravir has been approved for the treatment of HIV-1 infection in patients who have not previously received any antiretroviral medication (Table 6). Moreover, bictegravir is recommended for HIV-1 infection in patients who have been on a consistent antiretroviral regimen for at least three months and are virologically suppressed (HIV-1 RNA 50 c/mL), without a history of treatment failure, and without known risk factors for developing resistance to the individual components of the drug. It is used along with emtricitabine and tenofovir. In the body, bictegravir is quickly absorbed, T_max_ = 2.0–4.0 h. It should not be taken by people who have liver problems. The most typical adverse effects are headache, nausea, and diarrhea [119]. 

#### 5.3.2. Cabotegravir

Cabotegravir is an “HIV-1 integrase inhibitor” used to treat and prevent HIV-1 infection (Table 6). Early studies on cabotegravir revealed that it had a lower oral bioavailability than dolutegravir; this finding led to the development of a cabotegravir formulation for long-acting monthly intramuscular injections. In 2021, the FDA approved cabotegravir with rilpivirine as a treatment for HIV-1 infection in people with virological suppression [133].

#### 5.3.3. Doravirine

As a non-nucleoside reverse transcriptase inhibitor for HIV-1, doravirine is used in conjunction with other antiretroviral drugs to reduce the body’s HIV burden and improve the function of the immune system (Table 6). Doravirine is officially indicated for the treatment of HIV-1 infection in adult patients who have never received antiretroviral therapy. For adult patients with HIV-1 infection who have never received antiretroviral therapy, doravirine is an appropriate choice in combination with other antiretroviral medications. Additionally, it is advised that patients who are virologically suppressed (HIV-1 RNA less than 50 copies per mL) and on a stable antiretroviral regimen without a history of treatment failure or mutations linked to doravirine resistance be switched to a new antiretroviral regimen. Doravirine has an absolute bioavailability of 64% and a T_max_ of 2 h. Throughout clinical tests, co-administration of doravirine with food did not significantly change its pharmacokinetic profile. Following intravenous administration, doravirine has a steady-state volume of distribution of 60.5 L. In plasma, doravirine is approximately 76% protein-bound. It has an elimination half-life of 15 h [134].

#### 5.3.4. Emtricitabine

Emtricitabine, a cytidine analogue, is a nucleoside reverse transcriptase inhibitor (NRTI) used to treat HIV infection in adults or to prevent HIV infection in high-risk adults and adolescents when coupled with tenofovir alafenamide (Table 6). it prevents HIV RNA from being converted to DNA by inhibiting HIV reverse transcriptase. Emtricitabine competes with the natural substrate of HIV-1 reverse transcriptase for incorporation into freshly synthesized DNA, thereby stopping transcription. It has a lengthy duration of action because it is injected only once per day. Patients need to be warned about the possibility of developing lactic acidosis and hepatomegaly with steatosis. Emtricitabine capsules have a bioavailability of 93%, while the oral solution has a bioavailability of 75%. The C_max_ of emtricitabine is reduced by 29% when taken with meals. Emtricitabine has an apparent central volume of distribution of 42.3 L and a peripheral volume of distribution of 55.4 L. Only 4% of emtricitabine is bound to serum albumin and other plasma proteins. Emtricitabine has a half-life of roughly 10 h [80].

### 5.4. FDA-Approved Fluorinated Antibacterial Drugs 

F-containing heterocyclic drugs have long been used as antibacterial agents. Fluoroquinolone antibiotics are the most well-known and widely utilized F-containing antibacterial antibiotics [82,86]. Fluoroquinolones have a broad antimicrobial spectrum. A F substituent significantly improves the antibacterial activity of the drug. Fluorinated antibacterial medicines have been produced to treat both novel and established bacterial strains.

#### 5.4.1. Berotralstat

Approved by the FDA in December 2020, berotralstat is recommended for the prevention of attacks of hereditary angioedema (HAE) (Table 7). This uncommon genetic condition causes significant swelling of the skin and upper airways. The production of excess bradykinin is regulated by the plasma kallikrein inhibitor berotralstat, which binds to plasma kallikrein and blocks its proteolytic activity during HAE attacks. CYP2D6 and CYP3A4 metabolize berotralstat. The metabolism and metabolites have not yet been defined for this drug. Following a single oral dose of 300 mg radiolabeled berotralstat, the unmodified medication accounts for about 34% of the total plasma radioactivity, whereas roughly eight identifiable metabolites contribute between 1.8 and 7.8% of the total radioactivity. After taking a single oral dose of 300 mg radiolabeled berotralstat, about 9% of the drug is excreted in urine, where the parent drug accounts for between 1.8–4.7% of the total radiolabeled molecule. Berotralstat frequently causes stomachache, heartburn, nausea, vomiting, diarrhea, and back pain. There is no information available regarding berotralstat overdose [89].

#### 5.4.2. Elexacaftor

Elexacaftor is a small-molecule cystic fibrosis transmembrane regulator corrector (CFTR corrector). Patients with cystic fibrosis and at least one F508del-CFTR mutation are treated with this drug in conjunction with tezacaftor and ivacaftor (Table 7). Elexacaftor is recommended for the treatment of cystic fibrosis (CF) in individuals 12 years of age and older who have at least one F508del mutation in the CFTR gene. It is used in conjunction with ivacaftor and tezacaftor as the combination product Trikafta^TM^. Elexacaftor functions as a CFTR corrector to enhance the number of mature CFTR proteins on cell surfaces. Drugs like elexacaftor help to reduce several multi-organ CF symptoms, including lung function, nutritional status, and general quality of life, when combined with CFTR potentiators, which improve the function of cell-surface CFTR proteins. Elevated liver transaminases can result from treatment with Trikafta^TM^, a triple combination drug that contains elexacaftor. Elexacaftor has an absolute oral bioavailability of about 80%. When administered at 200 mg once daily, elexacaftor has a median T_max_ of 6 h, and a steady-state AUC0–24h and C_max_ of 162 mcg/h and 8.7 mcg/h, respectively. It has an apparent volume of distribution equal to 53.7 L. In plasma, elexacaftor is >99% protein-bound, mostly to albumin. Elexacaftor undergoes substantial metabolism that is largely catalyzed by CYP3A4/5.6. Its primary active metabolite, M23-ELX, is just as potent as the original medication. In published studies, the specific metabolic route of elexacaftor has not yet been clarified. After administration of a radiolabeled dose of elexacaftor, around 87.3% is found in the feces, largely as metabolites, while just 0.23% is excreted in urine. Elexacaftor has a mean terminal half-life of about 24 h [135].

#### 5.4.3. Eravacycline 

As a member of the tetracycline class of antibiotics, eravacycline is a synthetic fluorocycline that has clinically significant efficacy against Gram-positive, Gram-negative, and facultative bacteria (Table 7). The affinity of this drug for human plasma proteins increases with plasma concentration, reaching between 79% and 90% (bound) at concentrations of 100 to 10,000 ng/mL. Eravacycline is metabolized predominantly by oxidation, which is mediated by CYP3A4 and FMO. Infusion site responses, nausea, and vomiting are the most typical adverse side effects (incidence 3%). Palpitations, chest pain, acute pancreatitis, pancreatic necrosis, hypocalcemia, dizziness, dysgeusia, anxiety, sleeplessness, depression, pleural effusion, dyspnea, rash, and hyperhidrosis are less frequent (incidence 1%) negative side effects [136].

#### 5.4.4. Pretomanid

Pretomanid is one of three medications that make up a three-drug combination used to treat pulmonary tuberculosis (Table 7). This medication has a history of myelosuppression, substantial hepatotoxicity, and lengthening of the cardiac QT interval. After a single 200 mg oral dose, the steady-state C_max_ of this drug was calculated to be 1.7 g/mL. The C_max_ of a 200 mg dosage measured in a different pharmacokinetic modeling study reported 1.1 g/mL. T_max_ was reached within 4 to 5 h in a study of healthy volunteers in a fed or unfed state. In the same study, the AUC was approximately 28.1 g.h/mL in the fasted state and approximately 51.6 g.h/mL in the fed state, demonstrating greater absorption of the drug when taken with high-fat food. Pharmacokinetic modeling calculated that pretomanid has a volume of distribution of 130 ± 5 L. Pretomanid binds to plasma proteins at a rate of about 86.4%. The metabolism of this drug is governed by several reductive and oxidative mechanisms; no single primary metabolic system has been found. Studies conducted in vitro show that CYP3A4 contributes 20% to the metabolism of pretomanid. In one pharmacokinetic study, a radiolabeled oral dose of 1100 mg of pretomanid was given to healthy adult male volunteers. It was found that, on average, 53% of the radioactive dosage was eliminated in urine. Metabolites in feces accounted for 38%. In the urine, an estimated 1% of the radiolabeled dose was found to contain unmodified parent compound. In a pharmacokinetic study of healthy subjects, pretomanid showed an elimination half-life of between 16–17 h [79].

#### 5.4.5. Telotristat Ethyl

Telotristat ethyl (TSE) is a prodrug of telotristat and is a tryptophan hydroxylase inhibitor. TSE is used to treat diarrhea caused by carcinoid syndrome in combination with somatostatin an. The FDA approved telotristat ethyl in March 2017 (Table 7). TSE from neuroendocrine tumors was found to be responsible for the treatment of diarrhea. Almost 92.8% of the TSE that is eliminated is found in feces and 0.4% is found in urine [88].

### 5.5. FDA-Approved Miscellaneous Fluorinated Drugs 

#### 5.5.1. Asciminib

Tyrosine kinase inhibitor (TKI) asciminib is used to treat chronic-phase myeloid leukemia (Ph^+^ CML), which has the Philadelphia chromosome. More specifically, it is an inhibitor of the ABL1 kinase activity of the BCR–ABL1 fusion protein. The activity of this kinase drives the proliferation of CML in the majority of affected patients. Additionally, asciminib has demonstrated efficacy in Ph^+^ CML with the T315I mutation, which results in a mutant BCR–ABL1. The ABL inhibitors currently available can be divided into those that target the active conformation of the kinase domain (dasatinib, bosutinib) and those that target the inactive kinase domain. These inhibitors compete at the ATP-binding sites of these proteins (imatinib, nilotinib, ponatinib). Asciminib is distinctive in that it functions as an allosteric inhibitor, attaching to the myristoyl pocket of the BCR–ABL1 protein and trapping it in an inactive configuration. The FDA approved Asciminib in October 2021 [92].

#### 5.5.2. Atogepant 

In September 2021, the FDA approved atogepant under the trade name Qulipta for the treatment of episodic migraine headaches (Table 4). Atogepant is a novel drug in that it is the first and only oral CGRP antagonist approved for preventative use in migraine headaches, despite two other members of the same drug family, namely ubrogepant and rimegepant, receiving prior approval. These agents are indicated only for abortive migraine therapy. Current practice guidelines advise the use of some anti-epileptic drugs (such as valproic acid or topiramate) or beta-blockers (such as propranolol) in patients needing preventative migraine therapy, all of which have substantial side effects. Atogepant is part of the “gepants” family of drugs, which are generally well tolerated and offer a desired therapy choice for patients experiencing negative side effects from other preventative medications [72].

#### 5.5.3. Avacopan 

In January 2022, the FDA approved avacopan for its use in combination with rituximab or cyclophosphamide for the treatment of adult patients with severe active granulomatosis polyangiitis (GPA) or microscopic polyangiitis (MPA), the two primary types of ANCA-associated vasculitis (Table 8) [117].

#### 5.5.4. Delafloxacin 

Delafloxacin is an antibiotic fluoroquinolone used to treat skin and skin structural infections (Table 8). The FDA approved delafloxacin in 2017 to treat acute bacterial skin diseases caused by bacterial infections. Delafloxacin stops bacterial DNA from functioning. Blood plasma proteins that specifically attach to serum albumin absorb this drug. The drug is eliminated in urine (65%) and feces (28%) [74].

#### 5.5.5. Elagolix

Elagolix, which has two extra fluoroaryl moieties in addition to a trifluoromethyl group, received FDA approval to treat endometriosis-related discomfort (Table 8). In phase 3 trials, elagolix reduced median estradiol concentrations in endometriosis patients in a dose-dependent manner to about 42 pg/mL for the 150 mg once-daily regimen and 12 pg/mL for the 200 mg twice-daily regimen. Additionally, 48 healthy adult premenopausal women participated in a randomized, placebo- and positive-controlled, open-label, single-dose, comprehensive crossover QTc study to examine the impact of elagolix on the QTc interval. Elagolix concentrations were seventeen times greater in subjects given a single dose of 1200 mg of the drug than in those given 200 mg of elagolix twice a day. However, there was no clinically significant lengthening of the QTc interval. Elagolix has a T_max_ of 1 h. The AUC and C_max_ may be decreased by up to 24 and 36%, respectively, after a high-fat meal (compared to fasting). There is evidence that elagolix is 80% bound to human plasma proteins. Elagolix has an estimated elimination half-life between 4–6 h [75].

#### 5.5.6. Enasidenib

Enasidenib is used to treat acute myeloid leukemia (Table 8). The medication is administered orally, especially to patients with refractory acute myeloid leukemia who have isocitrate dehydrogenase-2 gene-processing alterations. Only individuals with IDH mutant genes in their bone marrow and blood are eligible for this treatment. The small molecules inhibit cell proliferation and block the enzyme sites that lead to aberrant cell differentiation. Enasidenib was authorized by the US FDA in August 2017. An in vitro study demonstrated that enasidenib binds to plasma proteins. A high dose can cause the degeneration of the seminiferous tubule in males and females, hypospermia, atrophy of the prostate and seminal vesicles, an increase in the number of atretic follicles in the ovaries, and uterine atrophy [85].

#### 5.5.7. Lasmiditan

Lasmiditan is used to treat acute migraine headaches with or without aura (Table 4). This drug is rapidly absorbed when taken orally, with a median T_max_ of 1.8 h. An open-label study examining the pharmacokinetics of absorption reported a C_max_ and AUC of lasmiditan following oral administration of 322.8–122.0 ng/mL and 1892–746.0 ng/mL, respectively. According to reports, lasmiditan has a 40% bioavailability when taken orally. When taken with a high-fat meal, the C_max_ and AUC of the drug are increased by 22% and 19%, respectively, and the T_max_ is delayed by 1 h. These variations in absorption are quite small and are not anticipated to have an impact on clinical outcomes. About 55–60% of the plasma protein binding of lasmiditan is concentration-independent. Most non-CYP enzymes are involved in the hepatic and extra-hepatic metabolism of the drug, with ketone reduction being the main route. The particular metabolic enzymes of lasmiditan are still unknown, but according to FDA labeling, none of the following are involved in its metabolism: monoamine oxidases, CYP450 reductase, xanthine oxidase, alcohol dehydrogenase, aldehyde dehydrogenase, or aldo-keto reductases. Two of the metabolites of lasmiditan (M7 and M18) are thought to be pharmacologically inactive despite the fact that the metabolites of this drug have not been defined in published studies. Lasmiditan is largely removed through metabolism, with a tiny amount of its overall elimination through urine. About 66% of the modest amount of medication present in urine after a dosage comprises the S-M8 metabolite of the drug. Only 3% of a dose of lasmiditan is found unmodified in urine, suggesting that the drug undergoes significant metabolism before excretion. Lasmiditan has an average elimination half-life of 5.7 h [87].

#### 5.5.8. Lemborexant

Lemborexant is prescribed to treat insomnia that interferes with falling asleep and/or staying asleep (Table 8). Lemborexant has a volume of distribution of 1970 L, which indicates that it is widely distributed throughout tissue. It has a protein-bound percentage of about 94% in vitro, although the precise proteins to which it binds in plasma are still unknown. Lemborexant disposition animal models have shown that oral administration results in fast absorption of the drug. Lemborexant has a T_max_ of 1–3 h (3–5 h after administration of a high-fat and high-calorie meal). Increases in C_max_ and AUC of the drug occur at a rate that is marginally sub-proportional to the amount administered. In the presence of moderate hepatic impairment, the AUC, C_max_, and terminal half-life increase, while the AUC (but not half-life) is increased in the presence of mild hepatic impairment. Urine and feces contain 29.1% and 57.4% of the dosage, respectively. Less than 1% of the excreted amount found in the urine is the parent drug, which indicates significant metabolism. Lemborexant has a half-life of 17 and 19 h at dosages of 5 mg and 10 mg, respectively. Hemodialysis is likely to be ineffective in overdose circumstances since lemborexant is largely protein-bound [90]. 

#### 5.5.9. Lumateperone 

Lumateperone is used to treat both positive and negative symptoms in schizophrenia patients (Table 8). Its receptor binding profile is distinctive, and it varies from other antipsychotics in that it modifies the neurotransmitters glutamate, serotonin, and dopamine, which are all involved in the pathophysiology of schizophrenia. This novel antipsychotic has limited off-target activity in addition to being selective for dopamine (D2) receptors in the mesolimbic and mesocortical regions of the brain. Lumateperone is absorbed in small intestine and crosses the blood–brain barrier and, thus, has ability to penetrate the multidrug resistance protein 1 (MDR1). It has a T_max_ of 3–4 h after an oral dose. After being administered intravenously, lumateperone has a volume of distribution of about 4.1 L/Kg. Approximately 97.4% of the plasma protein is linked to lumateperone. Most unaltered lumateperone is eliminated in feces because of its molecular weight. Because the metabolites of lumateperone are highly water-soluble, they can be completely eliminated. lumateperone is excreted in urine in about 58% of cases and in the feces in about 29% of cases. According to reports, lumateperone has a half-life of 13–18 h [137].

#### 5.5.10. Melphalan flufenamide 

Melphalan flufenamide is a prodrug of melphalan used to treat relapsed or refractory multiple myeloma (Table 8). Melphalan flufenamide is more readily uptaken by cells than melphalan and it is cleaved to the active metabolite by aminopeptidases. Melphalan flufenamide was granted FDA approval in February 2021 but it was withdrawn from the market in the wake of the phase 3 OCEAN trial, which showed a reduction in overall survival in comparison to standard treatment with pomalidomide and dexamethasone, despite superior progression-free survival. Desethyl-melphalan and melphalan are produced through the metabolism of melphalan flufenamide. Monohydroxy and dihydroxy-melphalan are produced spontaneously during the hydrolysis of melphalan. There is little reliable information about how melphalan flufenamide is removed from the body. Studies on the mode of elimination are complicated by the rapid and spontaneous breakdown of free melphalan. However, this drug is anticipated to be eliminated largely through urine [97].

#### 5.5.11. Netupitant

A pro-drug version of netupitant, fosnetupitant, is used to stop nausea and vomiting caused by chemotherapy, in addition to other drugs (Table 8). The FDA approved it in 2018 as an alternative treatment option for patients suffering from chemotherapy-related nausea and vomiting. In healthy people and patients, the mean SD volume of distribution of fosnetupitant was 124 +/− 76 L and 296 +/− 535 L, respectively. In all species, netupitant is very tightly (>99%) bound to plasma proteins. Netupitant is multi-exponentially excreted from the body [100].

#### 5.5.12. Pimavanserin

Pimavanserin is a typical antipsychotic drug (Table 8). When used in conjunction with serotonin, it combines inverse agonist and antagonist actions. In 2016, the FDA granted approval for this drug, recognizing that it is exclusively intended to treat neurological side effects like hallucinations and delusions. Continuous research has shown that pimavanserin does not interact metabolically with other hormones generated by the midbrain pituitary glands, such as dopamine, which transmits messages from the brain to the smooth muscles. This drug must be taken orally, and a daily dose of 34 mg is recommended. After 10 days of administration, it is eliminated by urine and feces only. Treatment with pimavanserin should be avoided for subjects with psychological disorders who have previously taken other psychotic-related drugs or any kind of antibiotic for an extended period and, thus, antibiotic interactions with pimavanserin may result in fatal diseases like cardiac arrhythmia or reduction in blood plasma levels [101].

#### 5.5.13. Selinexor

Selinexor was approved for the treatment of multiple myeloma in conjunction with bortezomib and dexamethasone (Table 8). High doses of bortezomib and dexamethasone chemotherapy, followed by an autologous stem cell transplant, are the usual treatments for multiple myeloma. Additionally, selinexor was given accelerated approval for the treatment of adult patients with diffuse large B-cell lymphoma (DLBCL). This drug has a mean C_max_ of 680 ng/mL and a mean AUC of 5386 ngh/mL after a single 80 mg dose. Taking selinexor with food, whether a high-fat or low-fat meal, causes the AUC to rise by about 15–20%, but this is not anticipated to have any clinically significant effects. This drug has a mean apparent volume of distribution of 125 L. A phase 1 study that looked at the impacts of diet and formulation revealed mean apparent volumes of distribution ranging from 1.9 to 2.9 L/kg. Selinexor has an average elimination half-life of 6–8 h [105].

#### 5.5.14. Tafenoquine

An antiparasitic medicine “Karintafel ^TM^” containing tafenoquine as an active pharmaceutical agent is used to treat and prevent vivax malaria (Table 8). According to in vitro studies, tafenoquine exhibits an average 50% inhibitory concentration of 0.436 mcg against blood stages of seven strains of *P. falciparum*. Tafenoquine has an IC_50_ ranging from 0.5 to 33.1 mcg, which is higher than that of primaquine in *P. falciparum* strains. Tafenoquine demonstrated a reduced transmission at dosages of more than 25 mg/kg in tests defining its ability to prevent transmission against the sporogonic stage of *P. vivax*. Tafenoquine has a large distribution volume of about 2560 L. It has an extremely high affinity for plasma proteins in humans, accounting for about 100% of its binding. Tafenoquine is slowly eliminated from the body after being broken down by several metabolic pathways, with renal clearance of the unaltered form being extremely low. Tafenoquine has a lengthy half-life of almost 14 days [109].

#### 5.5.15. Ubrogepant

Ubrogepant is used to treat acute migraines both with and without aura (Table 4). By inhibiting the activity of a crucial transmitter involved in the etiology of migraines, ubrogepant effectively cures migraine headache discomfort. After oral delivery, ubrogepant accomplished T_max_ between 0.7–1.5 h. When given after a high-fat meal, T_max_ is delayed by around 2 h, and C_max_ is decreased by 22% without having a substantial impact on the AUC. Across the full recommended dosage range, ubrogepant has dose-proportional pharmacokinetics. After oral administration, there is an apparent core volume of dispersion of about 350 L. In vitro, ubrogepant is 87% protein-bound, but the precise proteins to which it binds are still unknown. Ubrogepant is removed mostly by metabolism, which is primarily mediated by CYP3A4. The most prevalent circulating substances in human plasma are discovered to be two circulating glucuronide conjugates and the parent drug in its unaltered form. Following administration of a single oral dose to healthy subjects, approximately 42% of the dose is excreted unaltered in feces and 6% was eliminated without modification in urine. The primary route of elimination is fecal/biliary, while renal excretion is comparatively modest. Ubrogepant has an elimination half-life of between 5 and 7 h [114].

#### 5.5.16. Upadacitinib

Upadacitinib, an oral Janus kinase-1 (JAK-1)-selective inhibitor, is used to treat moderate to severe forms of rheumatoid arthritis, active psoriatic arthritis, ankylosing spondylitis, and severe atopic dermatitis (Table 8). Across the therapeutic dose range, upadacitinib exhibits a dose-proportional pharmacokinetic profile. Following numerous once-daily injections, steady-state plasma concentrations of upadacitinib are attained in 4 days with negligible accumulation.

Food consumption has no clinically significant impact on the AUC, C_max_, or C_min of_ upadacitinib in its extended-release formulation. Upadacitinib’s distribution volume in a patient with rheumatoid arthritis with a body weight of 74 kg is predicted to be 224 L. The steady-state volume of distribution in a pharmacokinetic study of healthy volunteers taking the extended-release formulation was 294 L. Following administration of the extended-release formulation, the mean terminal elimination half-life of upadacitinib ranged from 8–14 h. In clinical trials, upadacitinib is cleared from systemic circulation by 90% within 24 h of treatment [120].

#### 5.5.17. Vericiguat

Vericiguat is a soluble guanylate cyclase (sGC) used to treat systolic heart failure and lower mortality and hospitalization rates (Table 8). sGCs are intracellular enzymes present in vascular smooth muscle cells (among other cell types) that catalyze the synthesis of cyclic guanosine monophosphate (cGMP) in response to activation by nitric oxide. They are an essential part of the NO–sGC–cGMP signaling pathway, which facilitates the regulation of the cardiovascular system. These diverse cellular effects have linked deficiencies in cyclic GMP production (primarily due to insufficient nitric oxide bioavailability) to the pathogenesis of various cardiovascular diseases. Vericiguat, manufactured by Merck under the trade name Verquvo, was given FDA approval in January 2021 for use in a subset of patients with systolic heart failure [121,138].

## 6. Conclusions

The fluorine editing of drug candidate molecules is regarded as one of the most promising strategies in the development of modern pharmaceuticals. During the period of 2016–2022, the FDA approved 54 drugs with F-containing groups. A large share among these fluoro-pharmaceuticals comprise fluorinated azine derivatives with other five- and six-membered heterocyclic rings. These drugs are being used in therapeutic fields such as multiple myeloma, lymphoma, HIV, chronic heart failure, chronic myeloid leukemia, (ANCA)-associated vasculitis, migraines, and NSCLC. Among these approved drugs, five carry radioactive isotopes of F (F-18) which deliver characteristic features and offer serviceability in PET. Among the small-molecule drug candidates, a large number contain an F atom at the carbocyclic aromatic ring either in the form of a fluoro group, monofluoro-methyl, difluoro-methyl, or trifluoromethyl group, instead of a heterocyclic moiety, which is the common part of approved fluorinated drugs. In addition to F-containing small molecules, in recent years (2019–2022), the FDA has approved the following oligonucleotides: vutrisiran (polyneuropathy of hereditary transthyretin-mediated amyloidosis in adults); inclisiran (atherosclerotic cardiovascular disease and familial hypercholesterolemia); lumasiran (hyperoxaluria type 1); and givosiran (acute hepatic porphyria). This review has covered the biological aspects of FDA-approved drugs, their doses, distribution, and removal. Indeed, the continuous development of the F methodology and a greater understanding of the effect of F on bio-properties are expected to provide strong support for fluorinated drugs in the future.

## Figures and Tables

**Table 1:** FDA-Approved Fluorinated (F-18) Radiolabeled Drugs.

Name	Structure	Mode of Action/Uses	References
Flortaucipir F-18	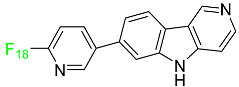	Small lipophilic tracer containing ^18^F capable of crossing the blood–brain barrier and binding to aggregated tau proteins; used in PET imaging for the diagnosis of Alzheimer’s disease.	[43]
Fluciclovine F-18	** 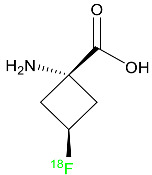 **	Transported into prostate cancer cells via ASCT2 and LAT1 transporters.	[44]
Fluorodopa F-18	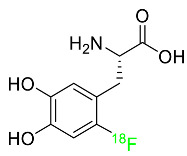	Decarboxylated by aromatic amino acid decarboxylase (AADC) in the striatum to fluorodopa F-18. Fluorodopa F-18 is then further metabolized by monoamine oxidase (MAO) to yield ^18^F 6-fluoro-3,4-dihydroxyphenylacetic acid.	[45]
Fluoroestradiol F-18	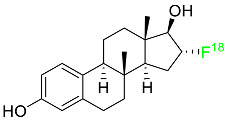	Radioactive diagnostic agent for the detection of estrogen receptor-positive lesions.	[46]
Piflufolastat F-18	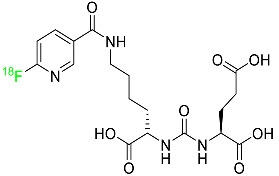	Binds to PSMA and allows for the visualization of PSMA-positive lesions associated with prostate cancer.	[47]

**Table 2:** FDA-Approved Fluorinated Oligonucleotide Drugs.

Name	Mode of Action with Therapeutic Uses	References
Givosiran	5-aminolevulinic acid synthase-directed small interfering RNA (siRNA) used in the prophylaxis of acute hepatic porphyria.	[51]
Inclisiran	PCSK9-targeting siRNA that lowers plasma LDL cholesterol levels.	[49]
Lumasiran	siRNA that silences hydroxyacid oxidase 1 for the treatment of primary hyperoxaluria type 1.	[50]
Vutrisiran	Transthyretin-directed siRNA used to treat polyneuropathy associated with hereditary transthyretin-mediated amyloidosis.	[48]

**Table 3:** FDA-Approved Fluorinated Heterocyclic/Carbocyclic Drugs.

Name	Structure	Mode of Action	References
Abemaciclib	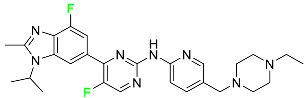	CDK4/cyclin D1 complex inhibitor	[70]
Alpelisib	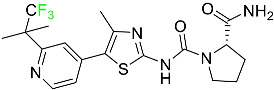	Phosphatidylinositol 3-kinase (PI3K) inhibitor	[71]
Atogepant	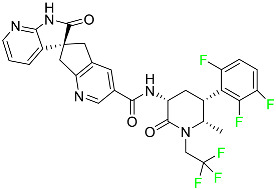	CGRP receptor antagonist	[72]
Apalutamide	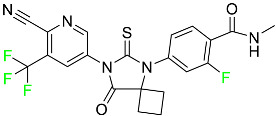	Androgen receptor inhibitor	[73]
Asciminib	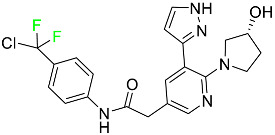	ABL/BCR–ABL1 tyrosine kinase inhibitor	[74]
Avacopan	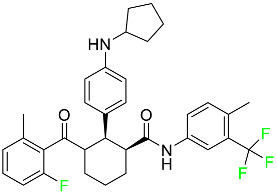	5a receptor (C5aR) antagonist	[75]
Avapritinib	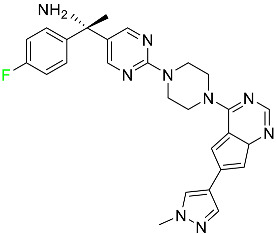	Selective tyrosine kinase inhibitor	[76]
Baloxavir marboxil	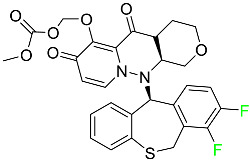	Endonuclease inhibitor	[77]
Belzutifan	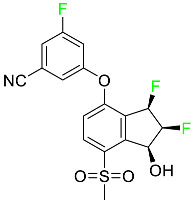	Hypoxia-inducible factor 2 inhibitor	[78]
Berotralstat	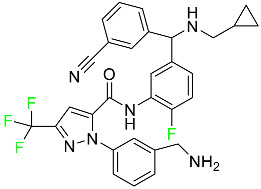	Plasma kallikrein inhibitor	[79]
Bictegravir	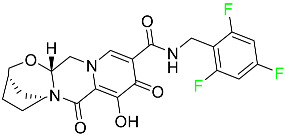	Integrase inhibitor	[80]
Binimetinib	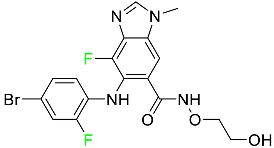	MEK inhibitor	[81]
Cabotegravir	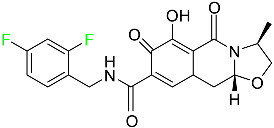	HIV-1 integrase inhibitor	[82]
Capmatinib	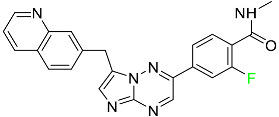	Kinase inhibitor	[83]
Dacomitinib	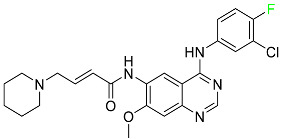	EGFR protein inhibitor, including EGFR/HER1, HER2, and HER4	[84]
Delafloxacin	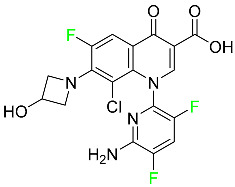	Bacterial DNA gyrase and topoisomerase IV inhibitor	[85]
Doravirine	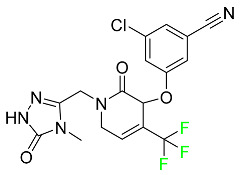	Non-nucleoside reverse transcriptase inhibitor	[86]
Elagolix	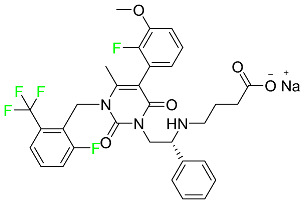	Gonadotropin-releasing hormone receptor antagonist (GnRH)	[87]
Elexacaftor	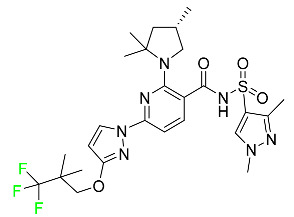	CFTR corrector	[88]
Emtricitabine	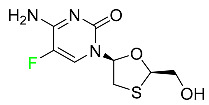	Nucleoside reverse transcriptase inhibitor (NRTI)	[89]
Enasidenib	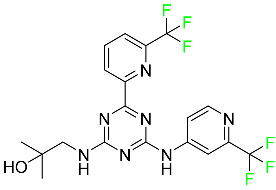	Isocitrate dehydrogenase-2 inhibitor	[90]
Encorafenib	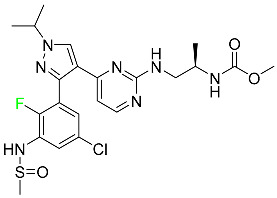	Kinase inhibitor	[91]
Eravacycline	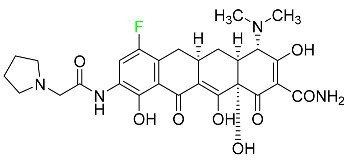	Binds to the bacterial ribosomal 30S subunit	[92]
Fostamatinib	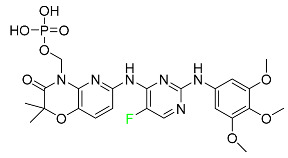	Tyrosine kinase inhibitor	[93]
Glecaprevir	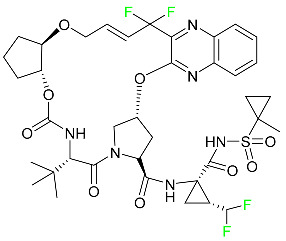	*NS3/4A and NS5A inhibitor*	[94]
Ivosidenib	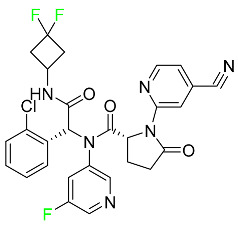	Isocitrate dehydrogenase-1 (IDH1) inhibitor	[95]
Larotrectinib	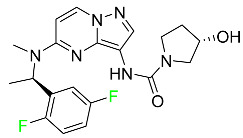	Tropomyosin receptor kinase (Trk) inhibitor	[96]
Lasmiditan	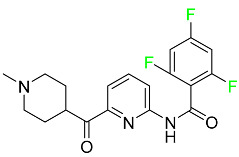	High-affinity serotonin (5-HT) 1F receptor agonist	[87]
Lemborexant	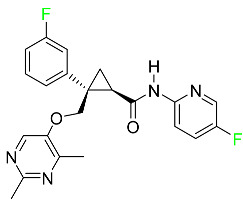	OX1R and OX2R antagonist	[97]
Letermovir	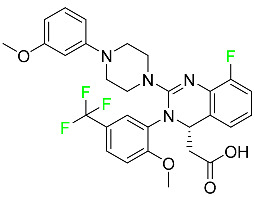	DNA terminase CMV complex inhibitor	[98]
Lorlatinib	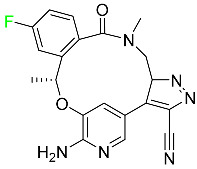	Tyrosine kinase inhibitor	[99]
Lumateperone	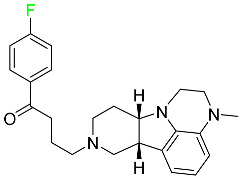	Receptor antagonist of 5-HT_2A_ receptor and antagonist of several dopamine receptors (D_1_, D_2_, and D_4_)	[100]
Melphalan flufenamide	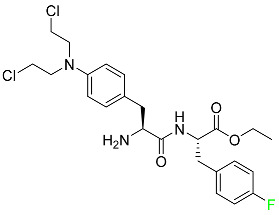	Peptidase-enhanced cytotoxic (PEnC)	[101]
Netupitant	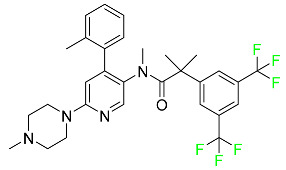	Selective neurokinin 1 (NK1) receptor antagonist	[100]
Osilodrostat	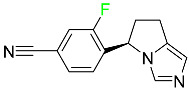	Cortisol synthesis inhibitor	[102]
Pexidartinib	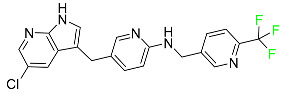	Tyrosine kinase inhibitor	[103]
Pibrentasvir	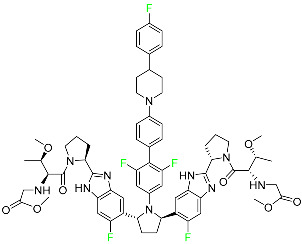	*NS3/4A and an NS5A inhibitor*	[94]
Pralsetinib	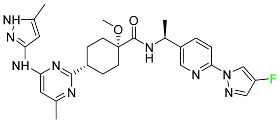	Kinase inhibitor	[104]
Pretomanid	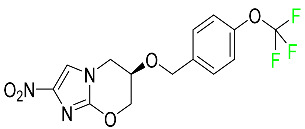	Inhibitor of cell wall biosynthesis via blockage of the oxidation of hydroxymycolate to ketomycolate	[72]
Pimavanserin	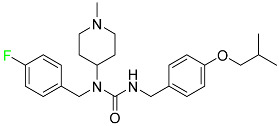	*Serotonin 5-HT2A receptor antagonist*	[105]
Relugolix	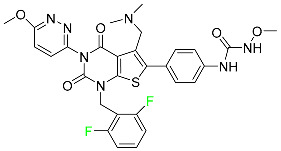	Nonpeptide GnRH receptor antagonist	[106]
Ripretinib	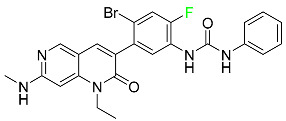	Protein kinase inhibitor	[107]
Rucaparib	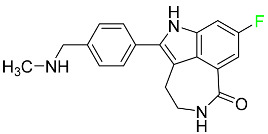	Polymerase inhibitor	[108]
Selinexor	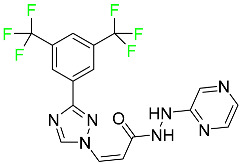	Selective nuclear transport (SINE) inhibitor	[109]
Selumetinib	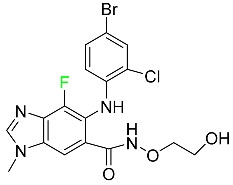	Selective MEK 1 and MEK 2 inhibitor	[110]
Siponimod	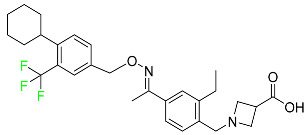	Sphingosine 1-phosphate receptor (S1P) modulator	[111]
Sofosbuvir	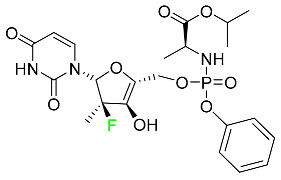	*Hepatitis C NS5B protein inhibitor*	[112]
Sotorasib	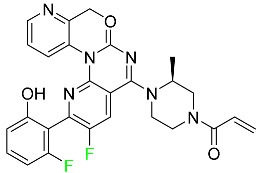	KRAS G12C inhibitor	[113]
Tafenoquine	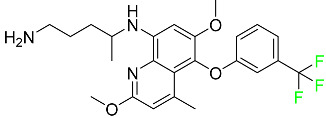	Antiparasitic agent	[114]
Talazoparib	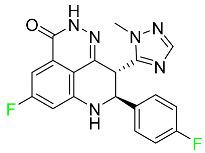	PARP1/2 enzyme inhibitor	[115]
Tecovirimat	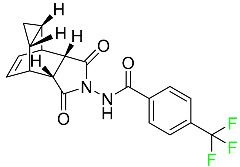	Orthopoxvirus VP37 inhibitor	[116]
Telotristat Ethyl	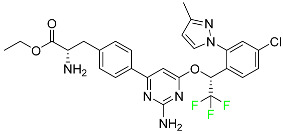	Tryptophan hydroxylase inhibitor	[117]
Tezacaftor	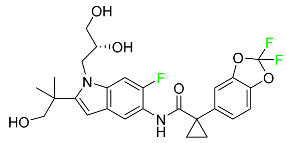	CFTR potentiator	[88]
Ubrogepant	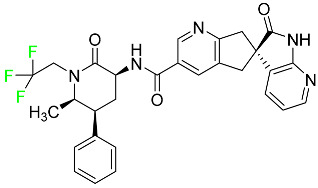	CGRP antagonist	[114]
Umbralisib	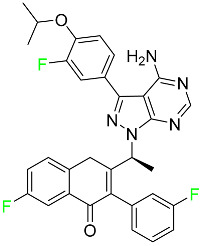	Kinase inhibitor	[118]
Upadacitinib	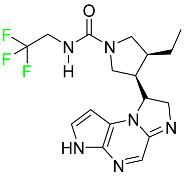	Selective Janus kinase-1 (JAK-1) inhibitor	[119]
Vericiguat	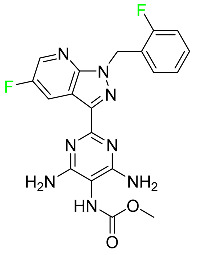	Guanylate cyclase (sGC) direct stimulant	[120]
Voxilaprevir	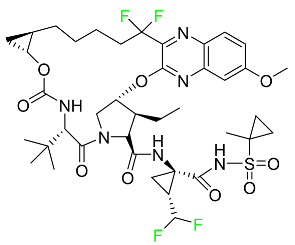	Protease inhibitor	[121]

**Table 4:** FDA-Approved Fluorinated Anticancer Drugs.

Name	Structure	Therapeutic Uses
Abemaciclib	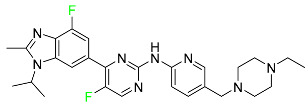	Solid tumors, including glioblastoma, melanoma, and lymphoma
Alpelisib	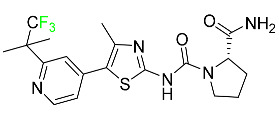	Strong anticancer efficacy
Apalutamide	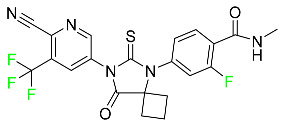	Non-metastatic castration-resistant prostate cancer
Avapritinib	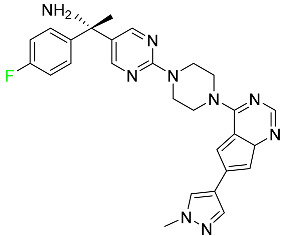	Gastrointestinal cancers that are resistant to many drugs
Belzutifan	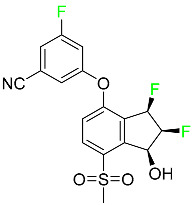	Antineoplastic for the treatment of some tumors connected to Von Hippel–Lindau (VHL) disease
Binimetinib	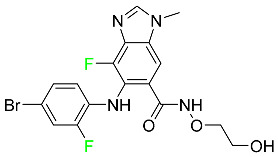	Chemotherapy drug with anti-tumor properties
Capmatinib	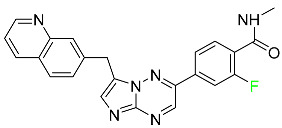	Non-small-cell lung cancer (NSCLC)
Dacomitinib	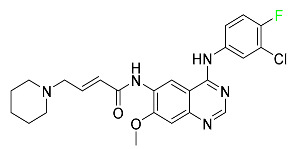	EGRF-mutated NSCLC
Encorafenib	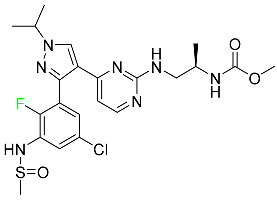	Metastatic melanoma
Ivosidenib	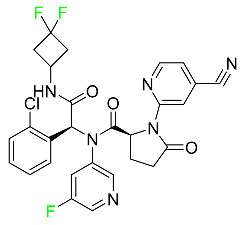	Malignancies with a sensitive IDH1 mutation
Larotrectinib	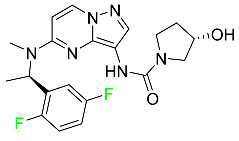	Solid tumors
Lorlatinib	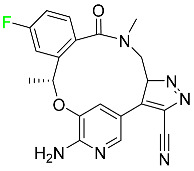	NSCLC
Pexidartinib	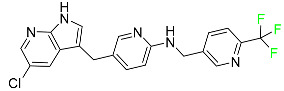	Tenosynovial giant cell tumor (TGCT)
Pralsetinib	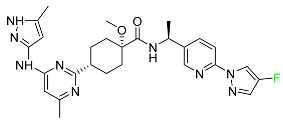	NSCLC
Relugolix	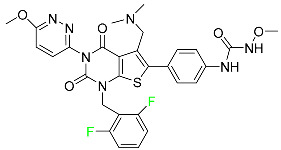	Prostate cancer
Ripretinib	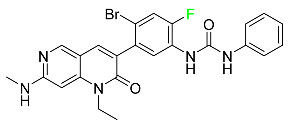	Gastrointestinal stromal tumors
Rucaparib	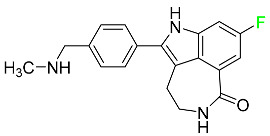	Adult patients with recurrent prostate and ovarian cancer
Selumetinib	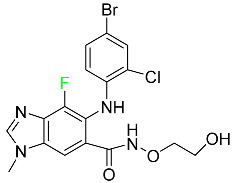	NSCLC
Sotorasib	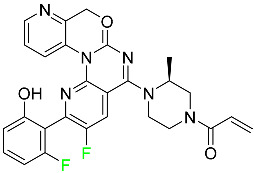	Mutant NSCLC harboring the KRAS G12C mutation
Talazoparib	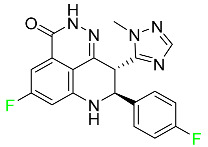	Germline BRCA-mutant, HER2-negative, locally advanced, or metastatic breast cancer

**Table 5:** FDA-Approved Fluorinated Anti-Viral Drugs.

Name	Structure	Therapeutic Uses
Baloxavir marboxil	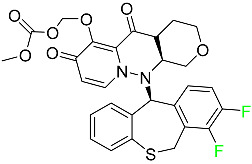	Influenza
Fostamatinib	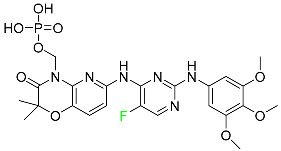	Acute respiratory distress syndrome (ARDS) in patients with severe COVID-19
Glecaprevir	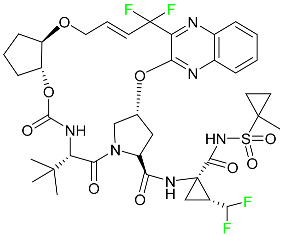	Chronic hepatitis C virus
Letermovir	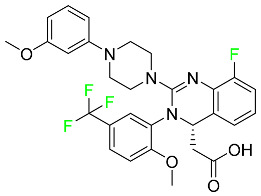	CMV infection as well as the prevention of infection following bone marrow transplantation.
Osilodrostat	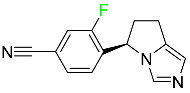	Cushing’s disease
Pibrentasvir	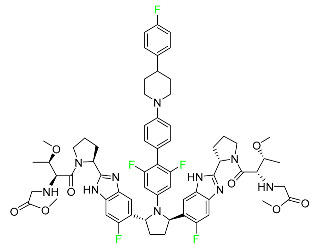	Chronic hepatitis C virus
Siponimod	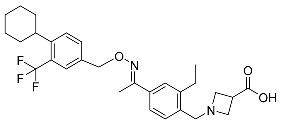	Multiple sclerosis
Sofosbuvir	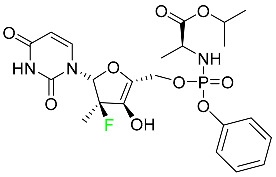	Hepatitis C in both adults and children older than 3 years
Tecovirimat	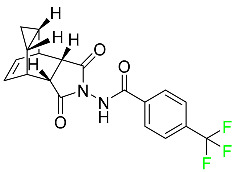	Human smallpox sickness in adults and children
Umbralisib	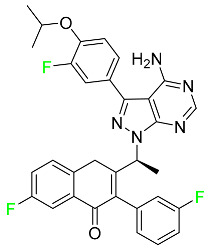	Non-Hodgkin lymphoma
Voxilaprevir	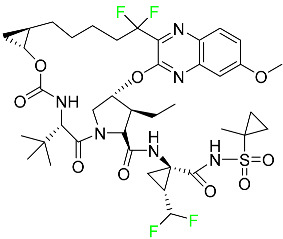	Hepatitis C infection

**Table 6:** FDA-Approved Fluorinated Anti-HIV Drugs.

Name	Structure	Therapeutic Uses
Bictegravir	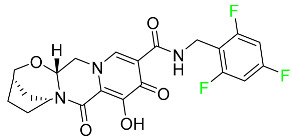	Treatment of HIV-1 infection
Cabotegravir	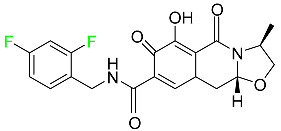	Treatment and prevention of HIV-1 infection
Doravirine	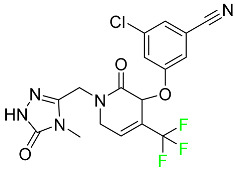	Treatment of HIV-1 infection in adult patients
Emtricitabine	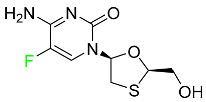	Treatment of HIV infection in adults and prevention of HIV infection in high-risk adults and adolescents when coupled with tenofovir alafenamide

**Table 7:** FDA-Approved Fluorinated Antibacterial Drugs.

Name	Structure	Therapeutic Uses
Berotralstat	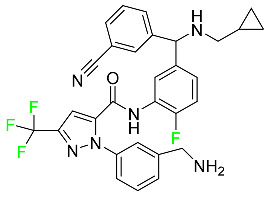	Prevention of attacks of hereditary angioedema (HAE)
Elexacaftor	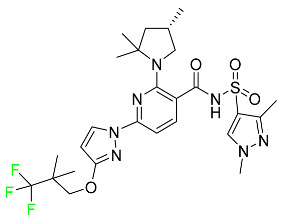	Treatment of cystic fibrosis
Eravacycline	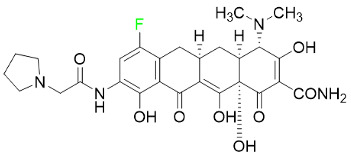	Antibiotic
Pretomanid	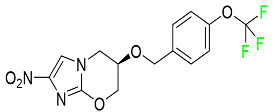	Treatment of pulmonary tuberculosis
Telotristat Ethyl	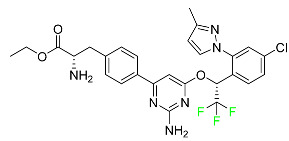	Treatment of diarrhea caused by carcinoid syndrome
Tezacaftor	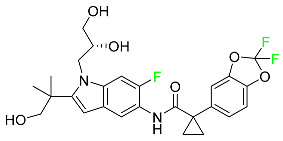	Treatment of cystic fibrosis

**Table 8:** FDA-Approved Fluorinated Miscellaneous Drugs.

Name	Structure	Therapeutic Uses
Asciminib	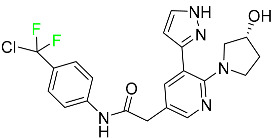	Chronic-phase chronic myeloid leukemia (Ph^+^ CML)
Atogepant	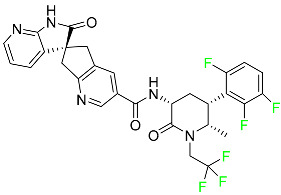	Episodic migraine headaches
Avacopan	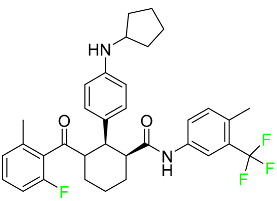	Granulomatosis polyangiitis (GPA) or microscopic polyangiitis
Delafloxacin	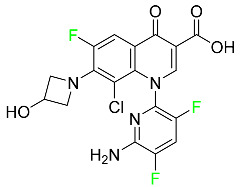	Skin structural infections
Elagolix	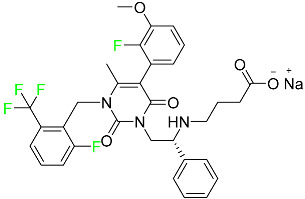	Endometriosis-related discomfort
Enasidenib	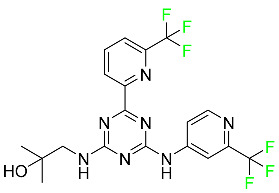	Acute myeloid leukemia
Lasmiditan	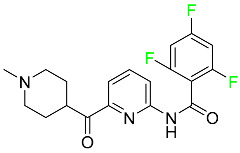	Acute migraine headaches with or without aura
Lemborexant	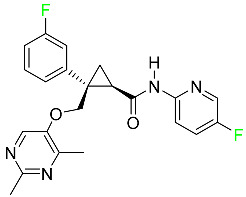	Insomnia that interferes with falling asleep and/or staying asleep
Lumateperone	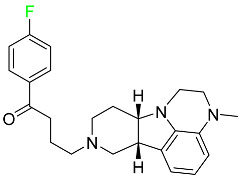	Positive and negative symptoms in schizophrenia patients
Melphalan flufenamide	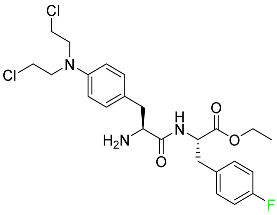	Relapsed or resistant multiple myeloma
Netupitant	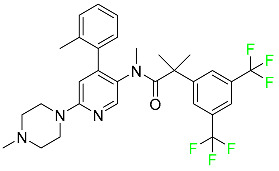	Nausea and vomiting brought on by chemotherapy in addition to other medications
Pimavanserin	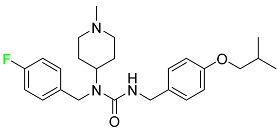	Anti-psychotic drug
Selinexor	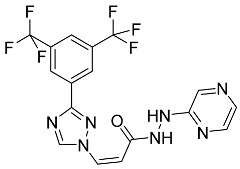	Multiple myeloma
Tafenoquine	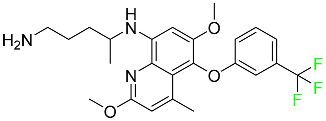	Treatment and prevention of vivax malaria
Upadacitinib	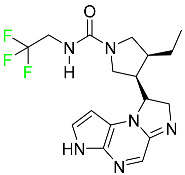	Moderate to severe forms of rheumatoid arthritis, active psoriatic arthritis, ankylosing spondylitis, and severe atopic dermatitis
Ubrogepant	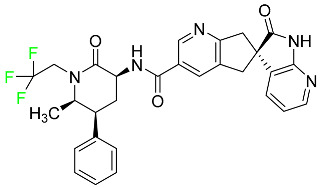	Acute migraines both with and without aura
Vericiguat	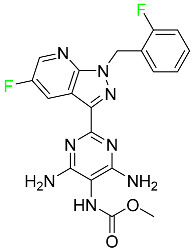	Systolic heart failure.

## Data Availability

Data sharing not applicable.

## Abbreviations

**S.NO.**	**Abbreviation**	**Explanation**
1	^18^F-DCFPyL	2-(3-{1-carboxy-5-[(6-[(18)F]fluoro-pyridine-3-carbonyl)-amino]-pentyl}-ureido)-pentanedioic acid,
2	ANCA	Antineutrophilic Cytoplasmic Antibodies
3	ASCT1	Alanine Serine Cysteine Transporter 1
4	AUC	Area Under the Curve
5	CDK4	Cyclin D and Cyclin-dependent Kinase 4
6	CFTR	Cystic fibrosis transmembrane conductance regulator
7	CGRP	Calcitonin Gene-related Peptide
8	CML	Chronic Myelogenous Leukemia
9	CMV	Cytomegalovirus
10	DLBCL	Diffuse large B-cell lymphoma
11	EGFR	Epidermal Growth Factor Receptor
12	eGFR	Estimated Glomerular Filtration Rate
13	EMBRACA	Epidemiological Study of Familial Breast Cancer
14	ER	Estrogen Receptor
15	FDA	Food and Drug Administration
16	FDG	^18^F-fluoro Deoxyglucose
17	GIST	Gastrointestinal Stromal tumors
18	GnRH	Gonadotropin-releasing Hormone
19	*HCV*	Hepatitis C Virus
20	HER1	Human Epidermal Growth Factor Receptor 1
21	HIF-2α	Hypoxia-Inducible Factor-2α
22	KRAS	Kirsten Rat Sarcoma Virus
23	KRAS	Kirsten Rat Sarcoma Viral Oncogene Homolog
24	LAT1, LAT3	L-type Amino Acid Transporters
25	MAO	Monoamine Oxidase
26	MEK	Mitogen-activated Protein Kinase
27	MET	Mesenchymal Epithelial Transition
28	NF	Neurofibromas
29	NFTs	Neurofibrillary tangles
30	NO–sGC–cGMp	Nitric oxide (NO)–NO-sensitive (soluble) guanylyl cyclase (sGC)–cyclic guanosine monophosphate (cGMP)
31	*NS3/4A*	A non-structural protein 3 and 4a protease inhibitor
32	OX1R	Orexin Receptor Type 1
33	PARP	Poly (ADP-ribose) Polymerase 1
34	PARP	Poly Adenosine Diphosphate-ribose Polymerase
35	PDGFRA	Platelet-derived Growth Factor Receptor Alpha
36	PET	Positron Emission Tomography
37	PSMA	Prostate-specific Membrane Antigen
38	RET	Rational Emotive Therapy
39	TNF	Tumor Necrosis Factor
